# Supplementary material for: Footprint of the host restriction factors APOBEC3 on the genome of human viruses
Source: PLoS Pathog. 2020 Aug 14;16(8):e1008718. doi: 10.1371/journal.ppat.1008718 (PMC7449416; doi:10.1371/journal.ppat.1008718)
Supplement: S1 Fig — A. A3G favors deamination of cytidine when preceded by another cytidine. The 5’CC dinucleotide motif is depicted in three possible codon contexts on both coding and template strand. Depending on the position of the mutated C, the C to T transition can be synonymous (S) or non-synonymous (NS). Proportion of S and NS mutations is reported when the two types of mutation can be produced. Because synonymous mutations are more likely to be retained, A3G-footprinted viruses should display to a stronger depletion of NCC codons compared to CCN or NNCCNN motifs (and/or a depletion of NNGGNN motifs versus the GNN and NGG motifs). B. The NCC and NNGGNN observed/expected ratios for 33,400 human viruses’ genomes (from 870 unique species) were calculated, grouped by species and colored according to the Baltimore classification. Each point represents a unique viral genome. Viral species with an NCC or NNGGNN ratio below two times the standard deviation (dotted grey line) from the population median (red line) are retained for further analysis in panel C. C. The observed/expected ratios of 5’CC dinucleotide at various codon positions and on both strands (i.e. NNCCNN, CCN, NCC, GGN, NGG and NNGGNN) were calculated for the NCC and/or NNGGNN depleted viral species and depicted by a heatmap. A colored scale with increasing shades of blue indicating depletion and increasing shades of red indicating enrichment. P-values were calculated by Student’s unpaired, two-tailed t-test (NS for not significant, * p< 0.05, ** p< 0.01, *** p< 0.001). D. List of the viral genes displaying NCC or NNGGNN depletion and belonging to an otherwise non-depleted viral genome. (PDF) [file ppat.1008718.s001.pdf]

Supplementary Figure 1

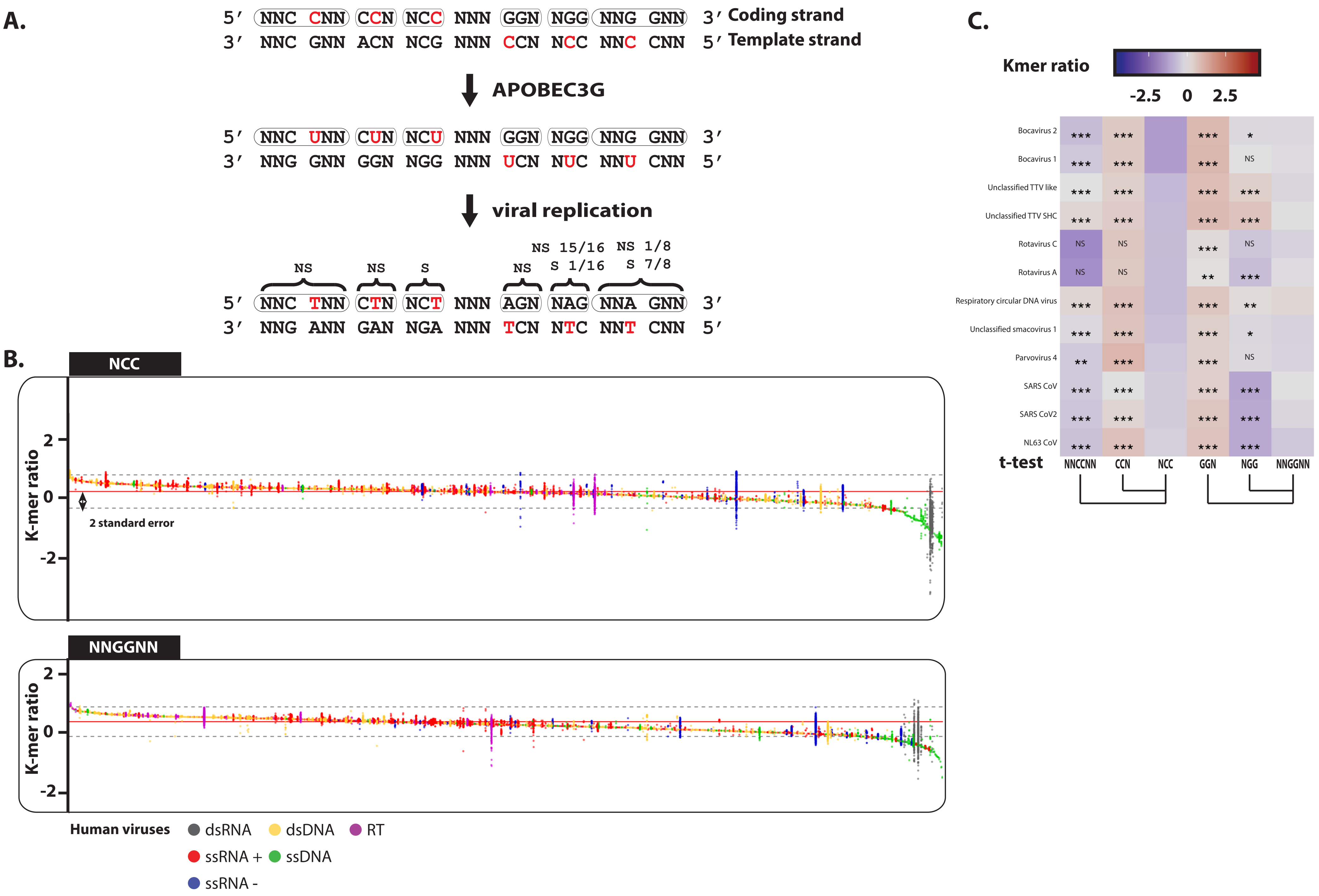

Supplementary Figure 1

D.

Group	Genus	Family	Specie	Gene	Genic NCC	Genic CCN	Genic NNCCNN	Genomic NCC
dsDNA	Herpesviridae	Varicellovirus	Alphaherpesvirus 3	ORF1	-0,82	0,40	-0,82	0,47
		Cytomegalovirus	Betaherpesvirus 5	US34A	-2,00	-0,42	-0,81	0,05
		Lymphocryptovirus	Gammaherpesvirus 4	BLLF2	-0,46	0,68	0,09	0,42
				BZLF1	-0,52	0,55	0,08	0,43
				LMP1	-0,79	-0,01	0,58	0,43
		Rhadinovirus	Gammaherpesvirus 8	K12	-0,73	0,90	0,58	0,28
				ORF30	-0,88	-0,28	0,71	0,28
	Papillomaviridae	Alphapapillomavirus	PV type 11	E4	-1,22	0,59	-0,22	0,27
				E7	-0,82	0,40	0,19	0,27
			PV type 18	E4	-1,48	0,08	-0,45	0,26
				E6	-1,63	-0,06	0,52	0,26
			PV type 30	E1	-0,66	0,65	-0,84	0,29
				E4	-0,88	1,07	-0,44	0,29
				E7	-0,63	0,17	0,25	0,29
			PV type 31	E4	-0,97	0,51	-0,13	-0,06
			PV type 33	E6	-1,70	0,92	-0,63	-0,01
			PV type 34	E1	-1,27	0,95	-0,88	-0,04
			PV type 35	E5	-1,32	-0,35	-1,30	-0,10
			PV type 45	E6	-0,75	-0,10	0,58	0,34
			PV type 51	E6	-1,33	0,57	-0,43	0,21
				E7	-1,82	0,54	0,21	0,21
			PV type 52	E4	-0,90	0,37	-0,61	0,13
				E5	-Inf	0,94	-0,60	0,13
				E7	-1,21	-0,64	-0,20	0,13
			PV type 53	E1	-0,80	0,78	-0,78	0,34
				E4	-1,01	1,16	-0,64	0,33
				E6	-2,11	0,21	-0,46	0,34
				E7	-2,25	0,37	0,33	0,34
			PV type 54	E4	-1,21	0,02	0,11	0,12
				E6	-1,54	0,75	0,40	0,12
			PV type 56	E1	-0,85	1,00	-1,06	0,13
				E6	-1,69	1,27	-0,65	0,13
				E7	-2,18	0,18	0,20	0,13
			PV type 58	E4	-1,69	0,49	-0,09	0,17
				E6	-1,40	0,90	-0,40	0,17
			PV type 61	E6	-1,00	0,43	0,09	0,41
			PV type 66	E1	-1,06	0,90	-0,54	0,13
				E7	-2,01	0,29	0,03	0,13
			PV type 67	E4	-0,72	0,28	0,18	0,26
			PV type 73	E1	-1,06	1,20	-0,66	-0,13
				L2	-1,21	1,29	-1,18	-0,13
			PV type 82	E7	-0,99	0,39	0,18	0,30
		Gammapapillomavirus	Gammapapillomavirus sp	E6	-1,08	0,66	-1,01	-0,06
	Polyomaviridae	Alphapolyomavirus	TS-associated PyV	Small T Ag	-0,67	0,61	1,10	0,23
		Betapolyomavirus	JC PyV	AGNO	-0,77	0,22	-0,76	0,52
		Deltapolyomavirus	MW PyV	VP3	-0,93	1,40	-0,96	0,07
		Orthopolyomavirus	Merkel cell PyV	Small T Ag	-2,43	0,58	0,57	0,45
				VP3	-1,23	-0,23	0,35	0,35
RT	Retroviridae	Deltaretrovirus	HTLV1	P12	-1,76	-0,08	0,18	0,32
		Lentivirus	HIV-1	REV	-0,78	0,62	0,25	0,17
			HIV-2	REV	-0,63	0,54	-0,29	0,24
ssRNA-	Paramyxoviridae	Rubulavirus	Mumps virus	SH	-0,88	-0,30	0,16	0,16
	Pneumoviridae	Orthopneumovirus	RSV	NS1	-1,40	-0,40	-0,42	0,20
				NS2	-0,76	0,45	-2,33	0,31
ssRNA+	Coronaviridae	Alphacoronavirus	NL63	M	-1,47	0,34	0,35	-0,34
				NS3	-1,53	0,42	-0,56	-0,34
			229E	E	-1,02	0,56	1,78	-0,16
				ORF4	-1,81	-1,24	0,67	-0,16
		Betacoronavirus	SARS2	E	-1,53	-0,54	-1,51	-0,39
				ORF10	-Inf	-0,26	-Inf	-0,39
				ORF7A	-2,49	0,09	-2,47	-0,39

Group	Genus	Family	Specie	Gene	Genic NNGGNN	Genic GGN	Genic NGG	Genomic NNGGNN
dsDNA	Herpesviridae	Varicellovirus	Alphaherpesvirus 3	ORF49	-0,61	0,59	-2,22	0,64
		Cytomegalovirus	Betaherpesvirus 5	UL139	-0,76	0,49	-0,09	0,38
				UL146	-0,50	0,94	-0,23	0,38
				UL15A	-0,76	0,24	-0,75	0,38
				UL19	-0,77	-1,53	-0,21	0,38
				UL2	-0,92	-0,10	-0,70	0,38
				UL9	-0,85	0,42	0,76	0,38
				US34A	-Inf	-Inf	0,55	0,38
				US6	-0,43	-0,29	-0,21	0,38
		Lymphocryptovirus	Gammaherpesvirus 4	A73	-0,18	-1,10	0,00	0,72
				LMP1	-0,08	1,22	-0,49	0,72
				RPMS1	-1,33	0,40	0,31	0,72
		Rhadinovirus	Gammaherpesvirus 8	K1	-0,29	0,48	-0,11	0,65
				K6	-1,01	-0,61	0,22	0,65
				K7	-0,33	0,22	0,54	0,65
	Papillomaviridae	Alphapapillomavirus	PV type 11	E4	-0,31	-1,33	0,20	0,60
				E5B	-0,43	0,29	-0,46	0,60
			PV type 18	E7	-0,88	-1,47	-2,47	0,41
			PV type 31	E6	-0,54	-0,04	-0,05	0,29
			PV type 33	E4	-Inf	-1,55	-0,56	0,31
			PV type 34	E7	-0,35	-1,37	-2,35	0,44
			PV type 35	E6	-0,94	-0,39	-0,16	0,44
			PV type 45	E5	-1,26	-Inf	-1,21	0,48
				E7	-0,42	-1,18	-1,86	0,50
			PV type 6	E5A	-0,19	-1,17	-0,20	0,59
				E5B	-0,32	0,67	0,11	0,59
			PV type 61	E4	-0,48	-0,50	0,12	0,73
			PV type 67	E4	-0,61	-0,22	0,10	0,39
				E7	-0,60	-0,21	-2,15	0,39
			PV type 73	E4	-1,44	0,11	-0,46	0,14
	Polyomaviridae	Betapolyomavirus	PyV 1	AGNO	-0,77	-0,15	-0,20	0,39
			JC PyV	AGNO	-0,46	-0,06	-0,47	0,44
ssDNA	Parvoviridae	Erythroparvovirus	Parvovirus B19	11 KDA	-1,32	-0,33	-Inf	0,19
ssRNA-	Filoviridae	Ebolavirus	Ebolavirus	VP30	-0,33	-0,09	0,03	0,40
	Pneumoviridae	Orthopneumovirus	RSV	NS2	-1,21	1,10	-0,24	-0,34
				M2	-0,99	-0,01	-0,59	-0,06
ssRNA+	Coronaviridae	Betacoronavirus	SARS2	M	-1,25	0,55	0,06	-0,22
				ORF7B	-Inf	-Inf	0,54	-0,22
