# Supplementary figures and images for: Footprint of the host restriction factors APOBEC3 on the genome of human viruses
Source: PLoS Pathog. 2020 Aug 14;16(8):e1008718. doi: 10.1371/journal.ppat.1008718 (PMC7449416; doi:10.1371/journal.ppat.1008718)

Supplementary  
Figure 2

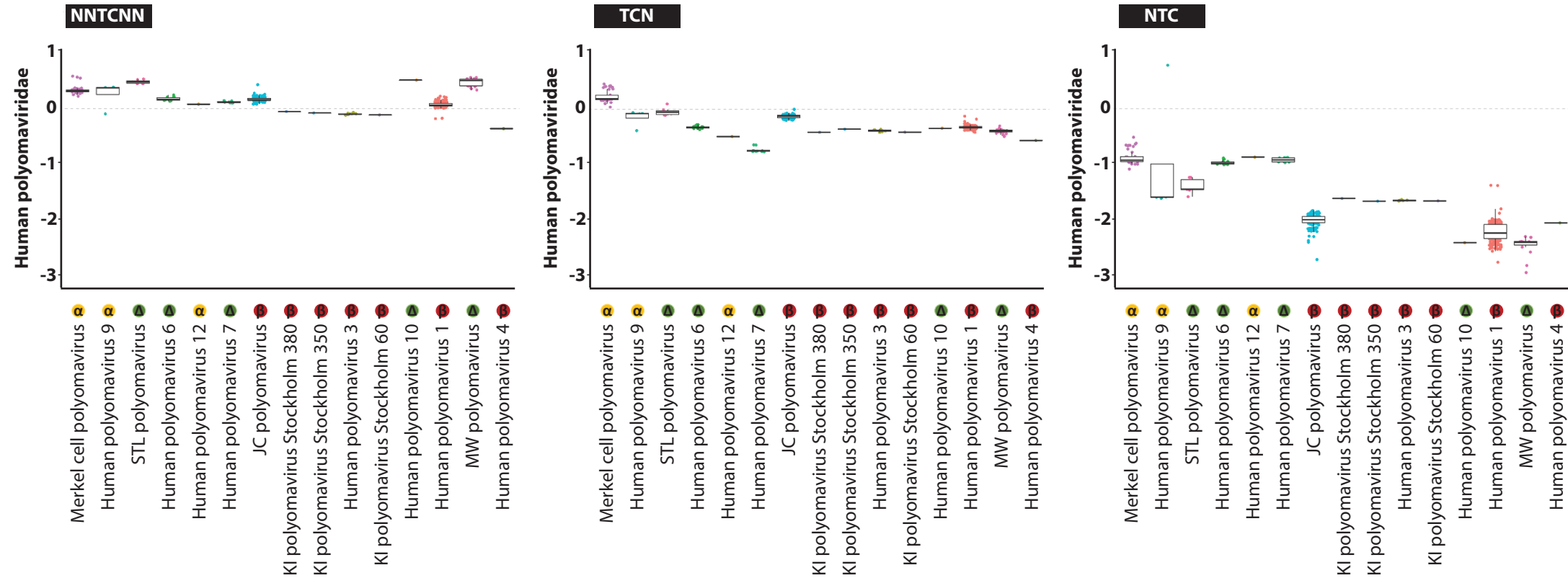

Supplement: S2 Fig — The observed/expected ratios of TC dinucleotide at various codon positions (i.e. NNTCNN, TCN, NTC) were calculated for several polyomaviruses and the corresponding genus (alpha, beta and delta) is reported for each virus. (PDF) [file ppat.1008718.s002.pdf]

### Supplementary Figure 3

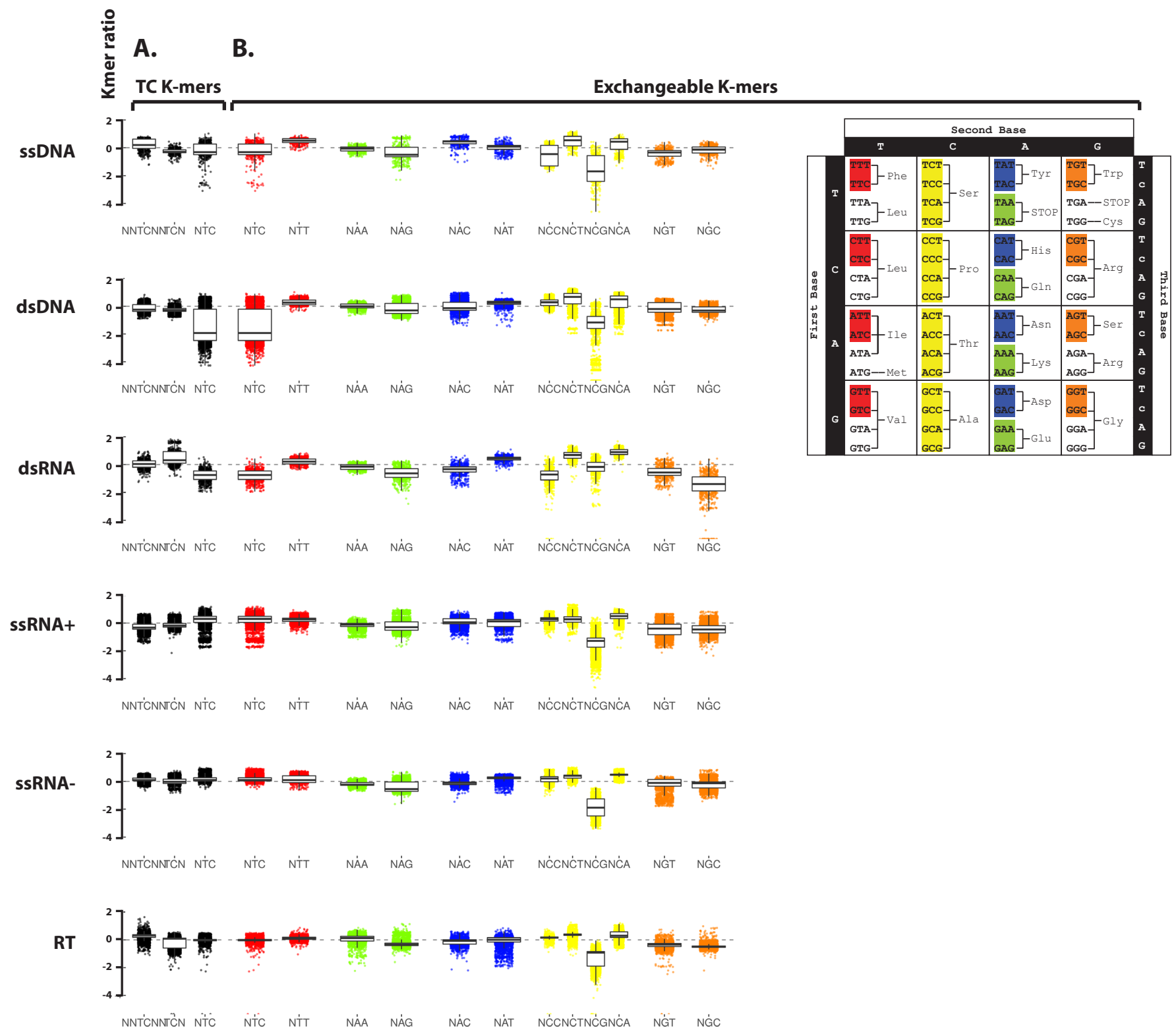

Supplement: S3 Fig — Human viruses were broken down into their respective Baltimore’s group and analyzed for their observed/expected K-mer ratios. (PDF) [file ppat.1008718.s003.pdf]

Supplementary  
Figure 4

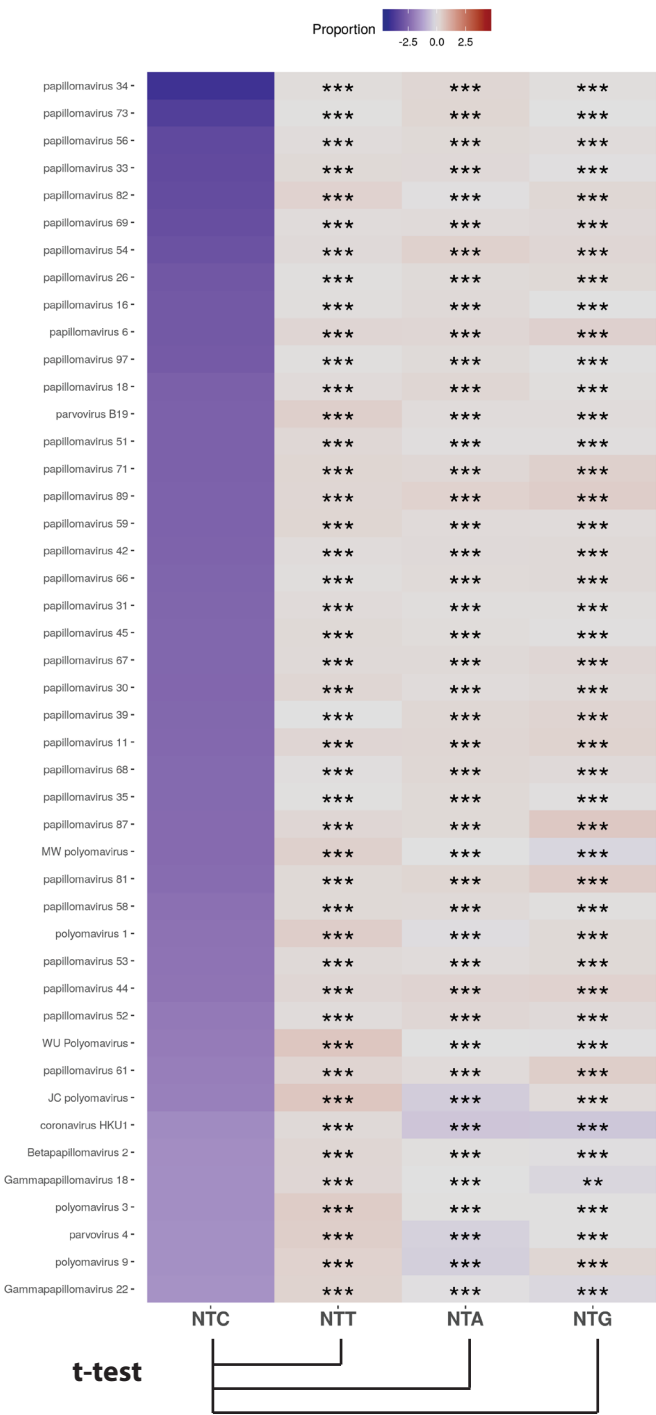

Supplement: S4 Fig — The observed/expected ratios of NTC, NTT, NTA and NTG K-mers were calculated for the putative A3-footprinted viral species and depicted by a heatmap. A colored scale with increasing shades of blue indicating depletion and increasing shades of red indicating enrichment. P-values were calculated by Student’s unpaired, two-tailed t-test (NS for not significant, * p< 0.05, ** p< 0.01, *** p< 0.001). (PDF) [file ppat.1008718.s004.pdf]

Supplementary  
Figure 5

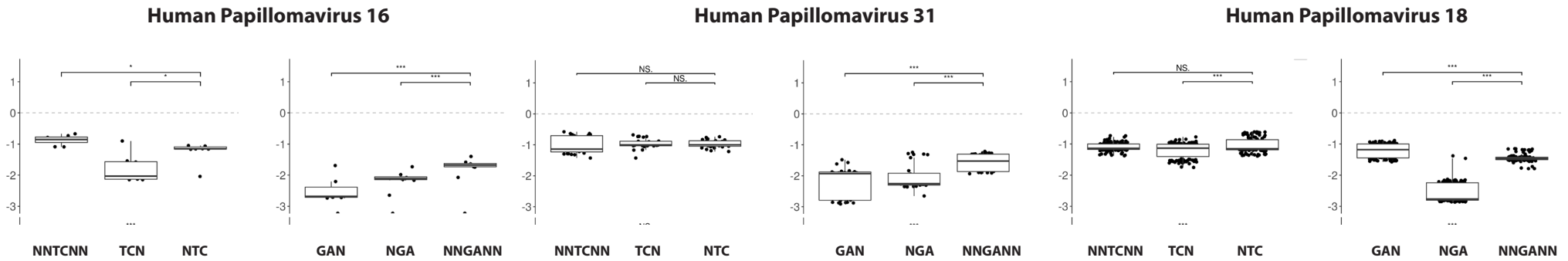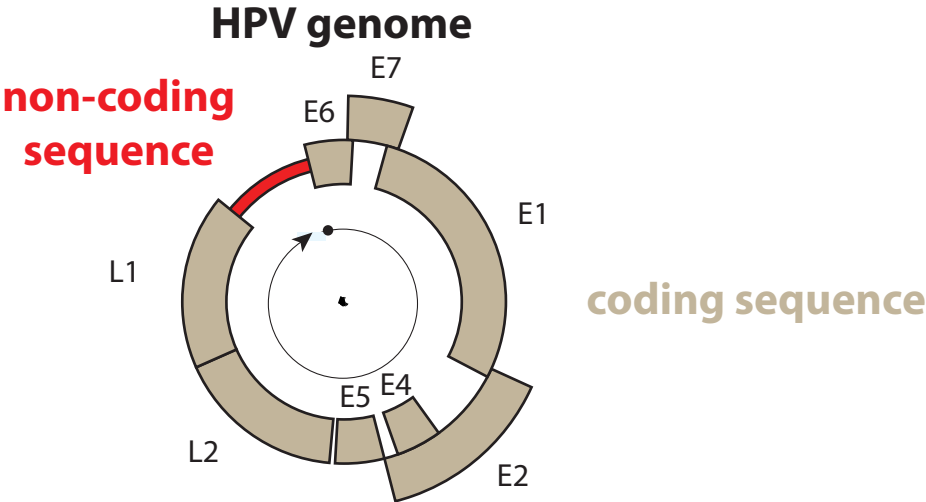

Supplement: S5 Fig — The observed/expected ratios of TC dinucleotide at various “codon” positions (i.e. NNTCNN, TCN, and NTC) were calculated for the non-coding sequences of human papillomavirus 16, 18 and 31. (PDF) [file ppat.1008718.s005.pdf]

Supplementary  
Figure 6

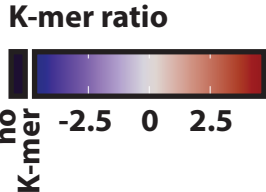

Alpha papillomavirus 16

Alpha papillomavirus 18

Alpha papillomavirus 31

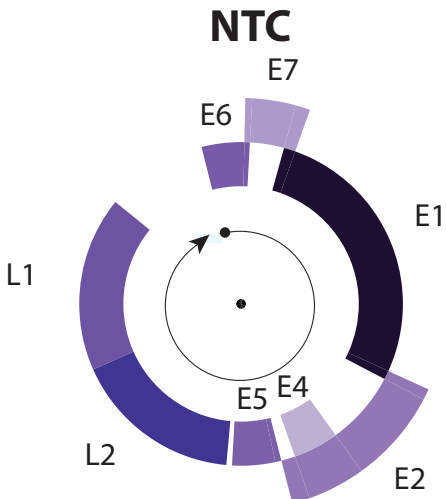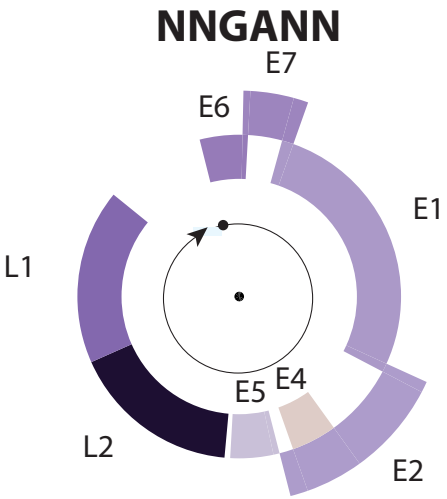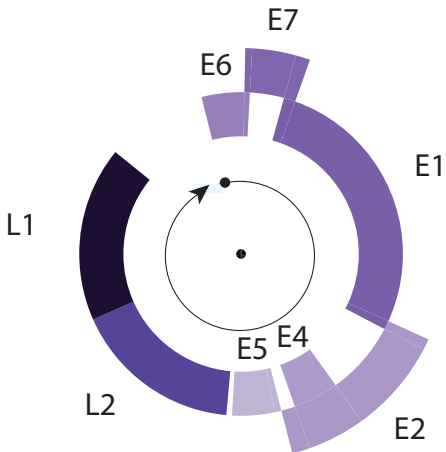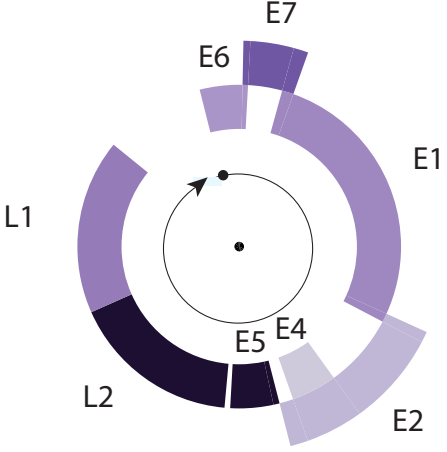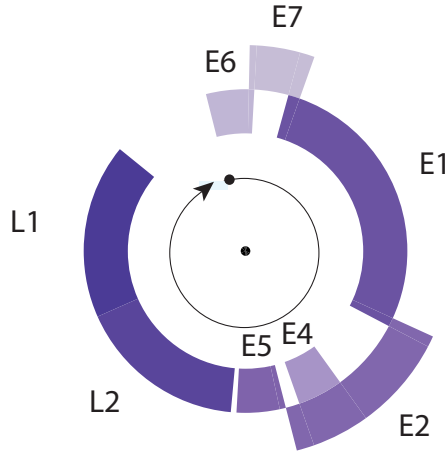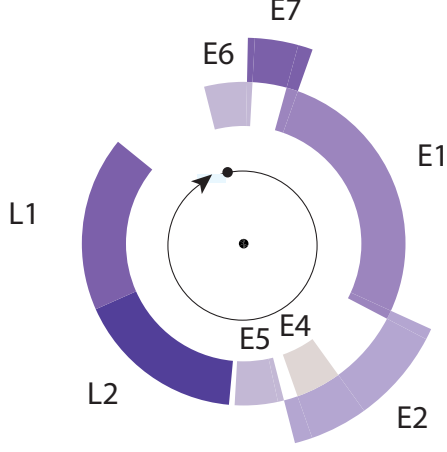

Supplement: S6 Fig — NTC and NNGANN observed/expected ratios were calculated for the different genes of the HPV16, HPV18 and HPV31 and were reported on their genomic maps using a colored scale with increasing shades of blue indicating NTC depletion and increasing shades of red indicating NTC enrichment. Replication origin is illustrated by a black dot and gene transcriptional orientation is symbolized by black arrow. (PDF) [file ppat.1008718.s006.pdf]

Supplementary  
Figure 7

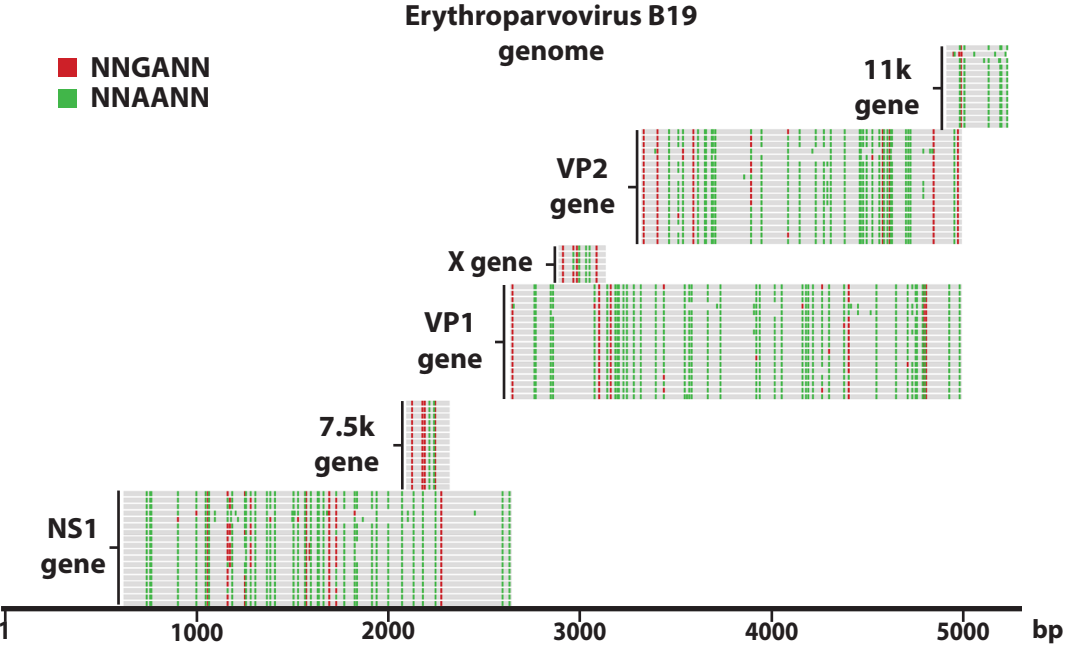

Supplement: S7 Fig — Coding sequences (NS1, 7.5k, VP1, X, VP2 and 11k genes) from 18 full-length B19 erythroparvoviruses were depicted by grey lines overlaid by red marks to symbolize NNGANN and green marks to position NNAANN codons. (PDF) [file ppat.1008718.s007.pdf]

Supplementary  
Figure 8

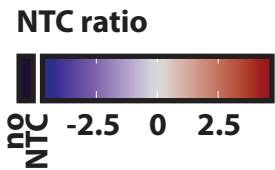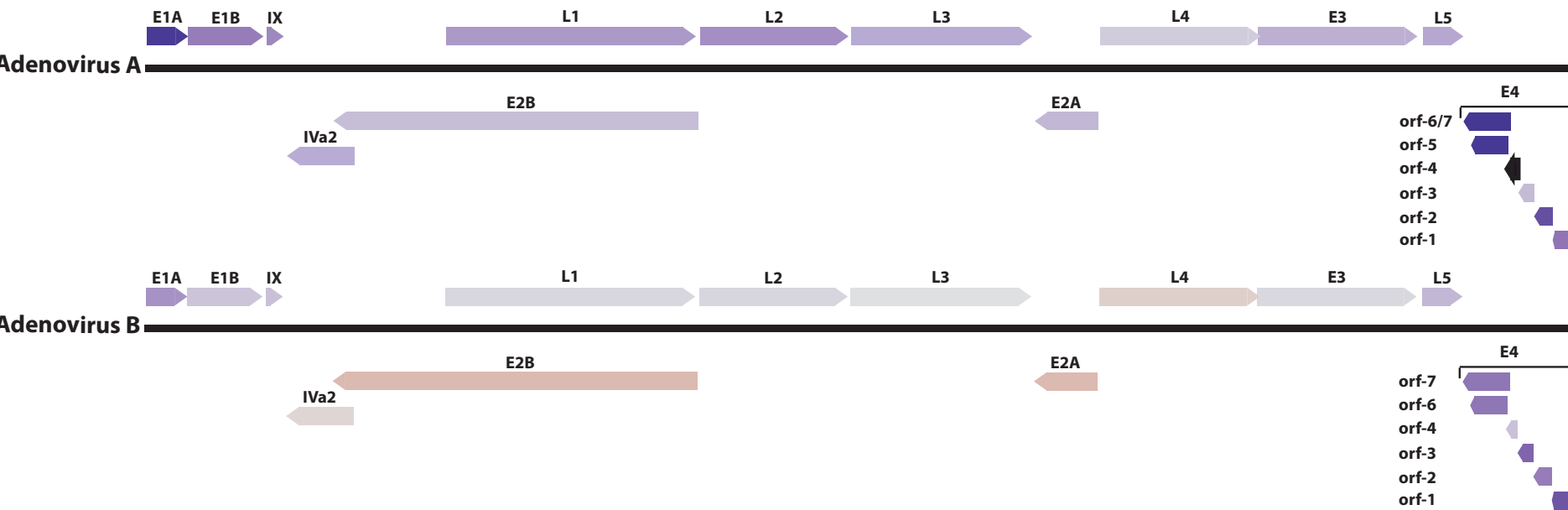

Supplement: S8 Fig — NTC observed/expected ratios were calculated for the different genes of the Adenovirus A and B and were reported on their genomic maps using a colored scale with increasing shades of blue indicating NTC depletion and increasing shades of red indicating NTC enrichment. (PDF) [file ppat.1008718.s008.pdf]

Supplementary  
Figure 9

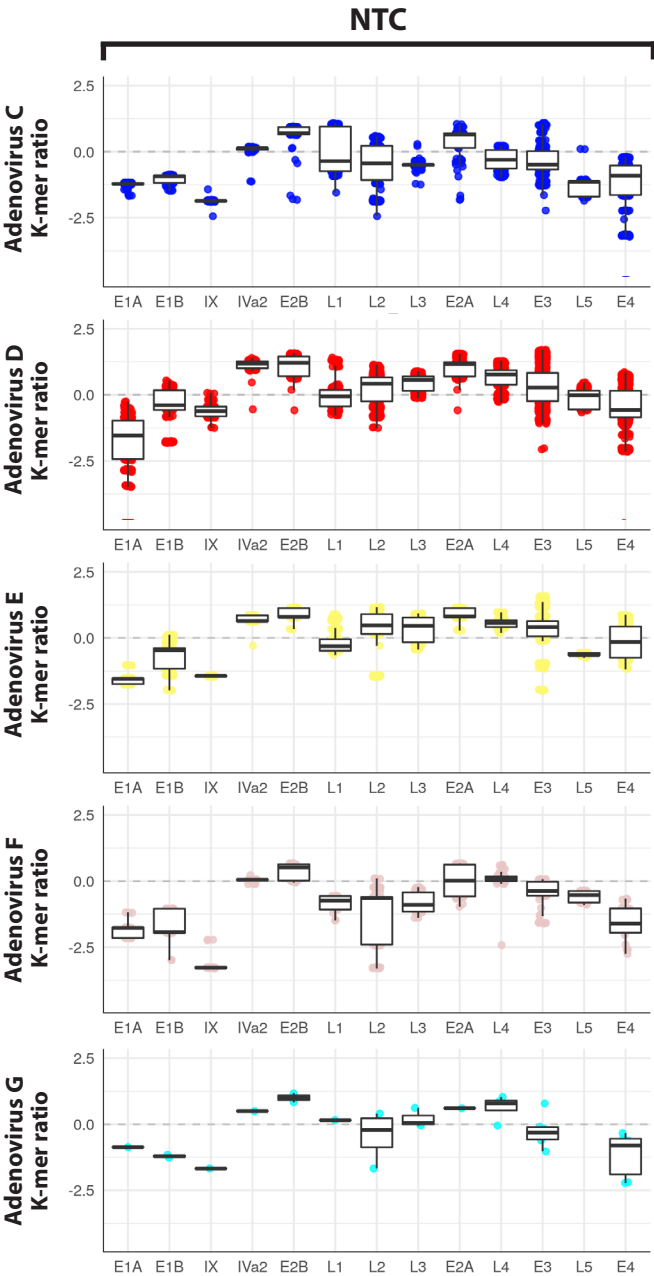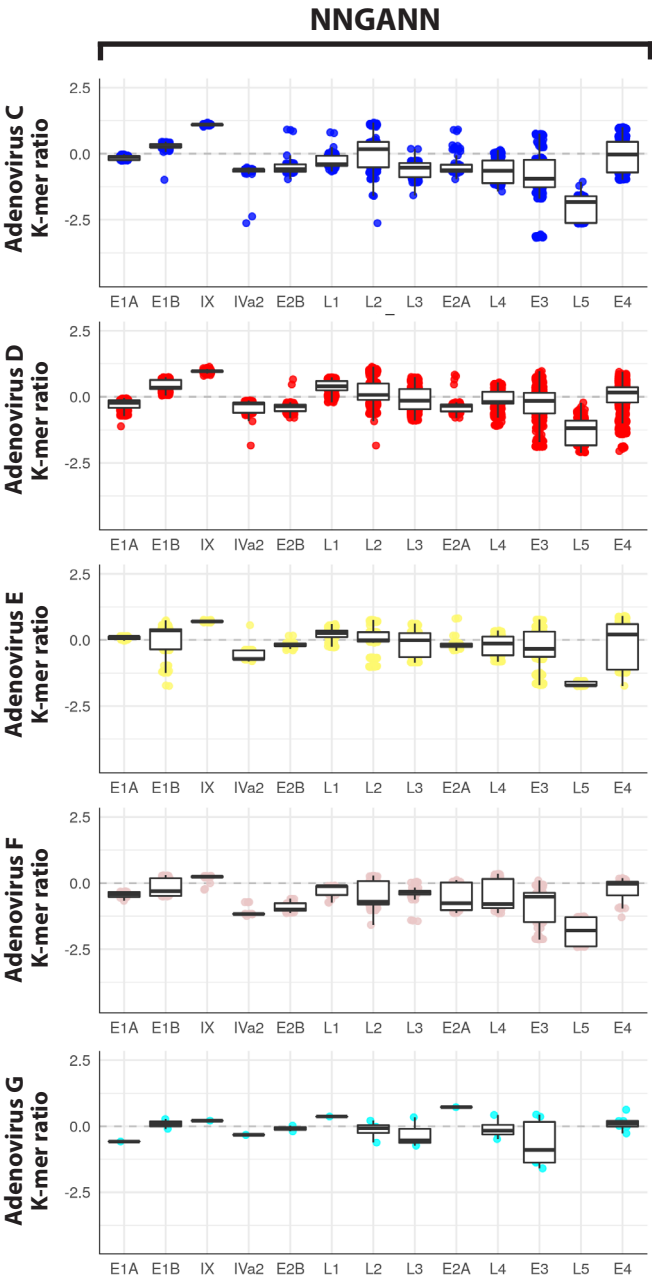

Supplement: S9 Fig — NTC and NNGANN observed/expected ratios were calculated for the different genes of the Adenoviruses C, D, F and G (each point represents a unique coding sequence). (PDF) [file ppat.1008718.s009.pdf]

Supplementary  
Figure 12

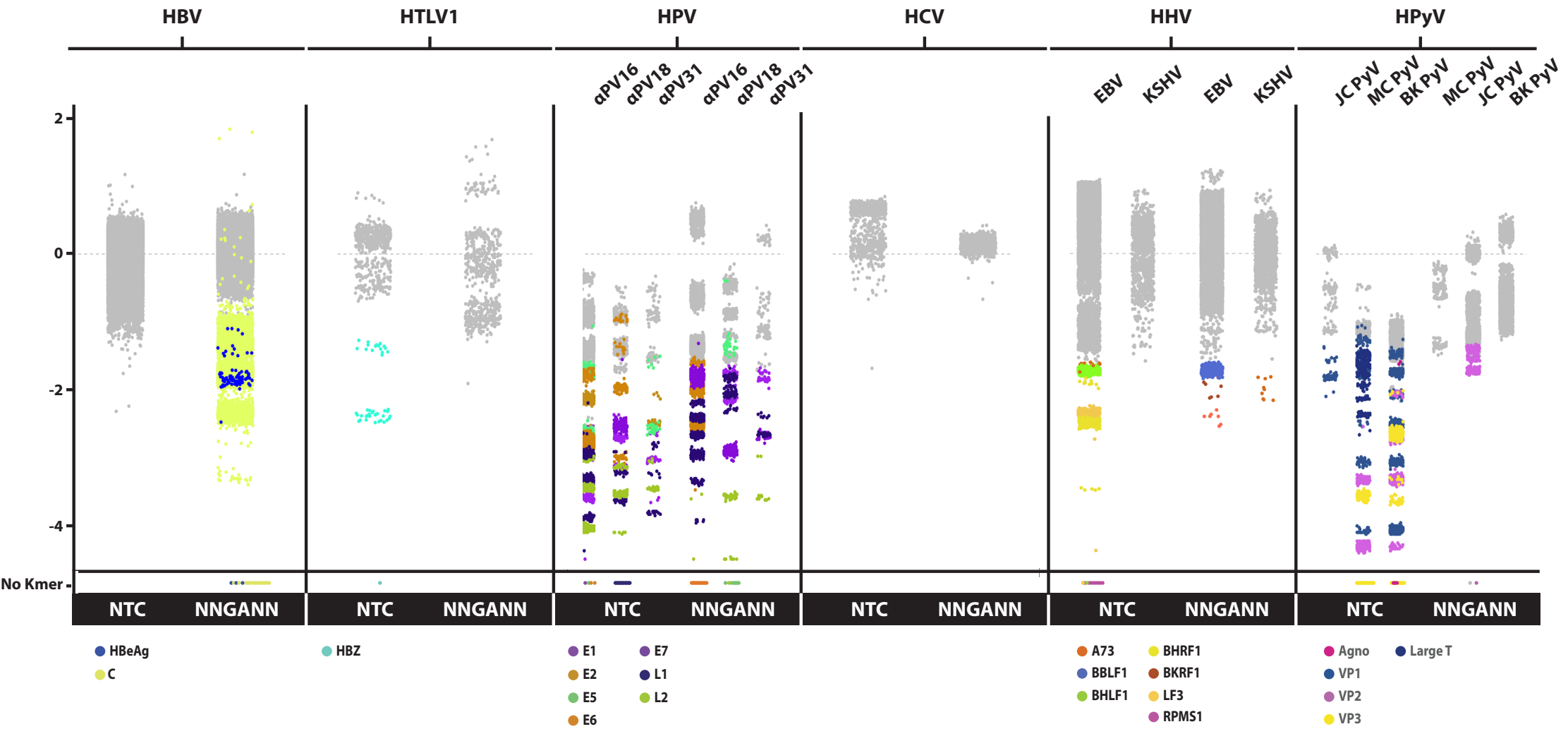

Supplement: S12 Fig — NTC and NNGANN observed/expected ratios were calculated for each available coding sequence of eleven well-known cancer-related viruses. Each point represents a unique viral coding sequence. The coding sequences are grouped and colored according to gene name. (PDF) [file ppat.1008718.s012.pdf]
