# Supplementary material for: Footprint of the host restriction factors APOBEC3 on the genome of human viruses
Source: PLoS Pathog. 2020 Aug 14;16(8):e1008718. doi: 10.1371/journal.ppat.1008718 (PMC7449416; doi:10.1371/journal.ppat.1008718)
Supplement: S10 Fig — A. AID favors cytidine deamination in a 5’ WRC context. The WRC trinucleotide motif is depicted in three possible codon contexts on both coding and template strand. Depending on the position of the mutated C, the C to T transition can be synonymous (S) or non-synonymous (NS). Proportion of S and NS mutations is reported when the two types of mutation can be produced. B. The WRC and NNGYWN observed/expected ratios for 33,400 human viruses’ genomes (from 870 unique species) were calculated, grouped by species and colored according to the Baltimore classification. Each point represents a unique viral genome. C. List of the putative AID-footprinted viral genes (displaying WRC or NNGYWN depletion) and belonging to an otherwise non-depleted viral genome. (PDF) [file ppat.1008718.s010.pdf]

Supplementary Figure 10

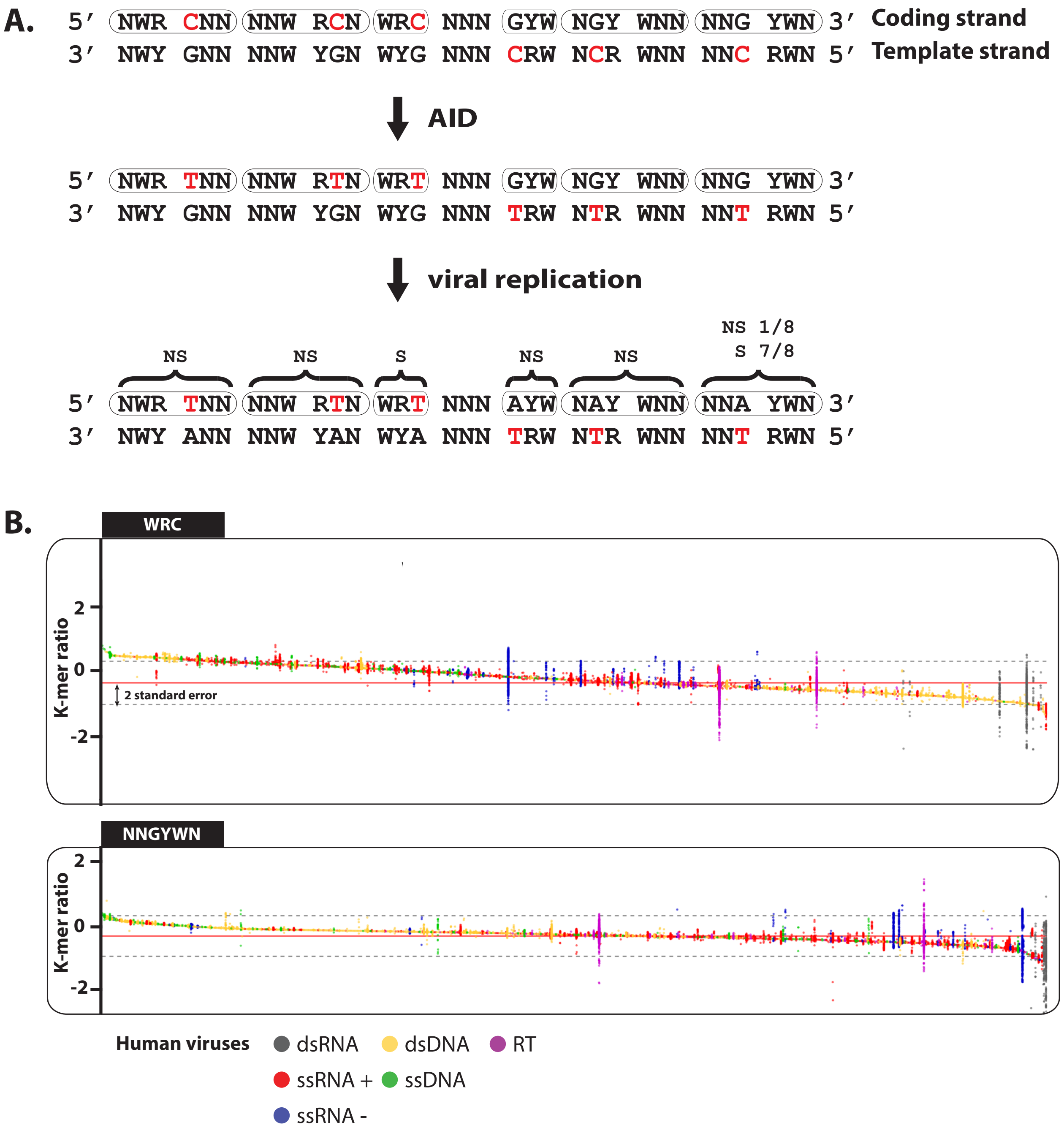

Supplementary Figure 10

C.

Group	Genus	Family	Specie	Gene	Genic NNGYWN	Genic NGYWNN	Genic GYW	Genomic NNGYWN
dsDNA	Herpesviridae	Simplexvirus	Alphaherpesvirus 1	UL14	-1,56	0,35	-0,28	0,65
				UL26,5	-1,64	0,39	-0,79	0,64
				UL8	-2,19	0,52	-0,58	0,65
			Alphaherpesvirus 2	UL23	-1,73	0,60	-0,89	0,64
				UL26	-1,60	0,61	-0,94	0,64
				UL43	-1,63	0,75	-1,27	0,64
				US10	-2,29	0,64	-2,33	0,64
		Varicellovirus	Alphaherpesvirus 3	ORF13	-1,85	0,00	0,07	0,65
				ORF50	-1,70	0,18	0,38	0,65
				ORF60	-2,35	0,24	0,40	0,65
				ORF13	-1,85	0,00	0,07	0,65
				GLYCOGL	-2,34	0,24	0,43	0,65
				GLYCOM	-1,70	0,16	0,38	0,65
		Cytomegalovirus	Betaherpesvirus 5	RL8A	-1,49	0,31	-0,98	0,93
				UL148C	-1,36	0,80	-0,38	0,93
				UL17	-1,66	0,17	-1,09	0,93
				UL96	-1,88	1,44	-0,38	0,93
				US33A	-1,95	1,50	-Inf	0,93
				US34A	-2,00	0,92	0,21	0,93
		Lymphocryptovirus	Gammaherpesvirus 4	BBLF1	-Inf	0,40	-Inf	0,65
				BBRF2	-1,69	0,45	-2,02	0,66
				BGLF3,5	-1,62	0,37	-0,24	0,68
				BLLF2	-1,76	-0,76	0,20	0,65
				BZLF1	-3,82	0,28	0,98	0,67
				LF3	-3,35	0,89	0,86	0,65
				LMP1	-1,57	0,06	-0,91	0,68
				UL14	-1,63	0,37	-0,22	0,65
		Rhadinovirus	Gammaherpesvirus 8	ORF23	-1,58	0,43	-0,48	0,67
				ORF73	-1,64	1,42	-0,56	0,67
	Papillomaviridae	Alphapapillomavirus	PV type 11	E4	-2,49	0,96	0,66	0,92
			PV type 30	E4	-1,56	0,52	0,14	0,99
			PV type 34	E5	-2,40	0,43	0,64	1,07
			PV type 35	E4	-1,31	0,02	1,14	1,19
			PV type 39	E4	-2,03	-0,44	0,78	1,09
			PV type 52	E4	-2,21	1,10	0,09	1,06
				E5	-1,67	1,49	-0,69	1,06
			PV type 53	E4	-1,86	0,15	0,22	1,04
			PV type 56	E4	-Inf	0,64	0,37	1,09
			PV type 6	E4	-1,42	0,75	0,89	0,94
			PV type 66	E4	-3,20	-0,03	0,25	0,98
		Gammapapillomavirus	PV type 172	E4	-1,27	0,43	-0,33	0,89
	Polyomaviridae	Deltapolyomavirus	PyV 7	VP2	-1,82	-0,50	0,62	0,63
				VP3	-2,08	-0,08	0,03	0,63
			MW PyV	VP3	-1,99	-2,06	0,85	0,72
dsRNA	Reoviridae	Rotavirus	Rotavirus A	VP2	-2,24	0,04	0,81	0,21
RT	Retroviridae	Lentivirus	HIV-1	VPU	-2,23	-0,24	1,25	0,70
			HIV-2	NEF	-1,57	0,31	-0,01	0,65
ssDNA	Parvoviridae	Dependoparvovirus	Adeno-associated virus	REP 40	-2,19	-0,24	-0,54	0,60
ssRNA-	Paramyxoviridae	Morbillivirus	Measles virus	C	-1,85	0,68	-0,16	0,70
		Respirovirus	Respirovirus 1	C	-3,37	-0,40	0,09	0,59
				Y1	-3,17	-0,41	0,30	0,59
				Y2	-3,10	-0,37	0,38	0,59
			Respirovirus 3	C	-3,25	-0,65	0,04	0,93
		Rubulavirus	Mumps virus	SH	-1,61	-0,06	-0,08	0,74
ssRNA+	Coronaviridae	Betacoronavirus	SARS2	ORF6	-Inf	-0,62	0,36	0,91
				ORF7B	-1,29	0,70	-1,33	0,91

Group	Genus	Family	Specie	Gene	Genic WRC	Genic NNWRCN	Genic NWRCNN	Genomic WRC
dsDNA	Adenoviridae	Mastadenovirus	Adenovirus B	E1A	-1,20	0,25	0,64	1,12
	Herpesviridae	Simplexvirus	Alphaherpesvirus 1	US11	-2,27	-0,46	0,73	1,24
				US8A	-1,56	-0,79	-1,13	1,24
			Alphaherpesvirus 2	US11	-3,20	-0,62	0,63	1,30
				US8A	-1,34	-0,57	-0,63	1,31
		Cytomegalovirus	Betaherpesvirus 5	UL17	-1,12	-Inf	-0,71	1,35
				UL21A	-1,34	-1,91	1,25	1,35
				UL30A	-1,22	-0,23	0,96	1,35
				US34A	-0,98	-1,91	1,48	1,35
		Lymphocryptovirus	Gammaherpesvirus 4	BGLF3,5	-1,25	-0,23	1,00	1,17
				BLRF2	-1,42	0,38	-0,10	1,06
				EBNA3B	-1,25	0,04	0,41	1,16
				UL14	-1,24	-0,24	1,01	1,06
		Rhadinovirus	Gammaherpesvirus 8	K12	-Inf	0,30	0,00	1,07
				ORF38	-2,16	-2,15	0,21	1,07
				ORF62	-1,48	-0,10	0,20	1,07
				ORF73	-1,60	-1,12	1,44	1,07
	Papillomaviridae	Alphapapillomavirus	PV type 11	L2	-2,24	1,08	0,22	0,67
			PV type 16	L2	-1,92	1,33	-0,16	0,62
			PV type 18	L2	-1,78	1,22	-0,30	0,66
			PV type 30	L2	-2,20	1,11	-0,08	0,66
			PV type 31	L2	-1,85	1,31	0,10	0,60
			PV type 34	L2	-1,83	1,21	0,35	0,64
			PV type 39	L2	-2,03	1,38	-0,14	0,55
			PV type 51	L2	-2,22	1,06	0,22	0,70
			PV type 53	L2	-3,20	1,05	-0,18	0,58
			PV type 56	L2	-2,75	1,23	-0,37	0,60
			PV type 6	L2	-1,67	1,05	0,03	0,68
			PV type 61	L2	-1,96	0,86	0,15	0,70
			PV type 66	L2	-1,77	1,21	-0,23	0,65
			PV type 67	L2	-2,52	0,96	-0,04	0,69
			PV type 68	L2	-1,82	1,42	0,00	0,58
			PV type 73	L2	-2,51	1,29	0,48	0,59
			PV type 82	L2	-1,66	1,16	0,30	0,75
	Polyomaviridae	Betapolyomavirus	PyV 1	AGNO	-1,90	0,90	0,45	0,61
		Deltapolyomavirus	MW PyV	VP2	-2,39	1,12	-0,74	0,51
RT	Hepadnaviridae	Orthohepadnavirus	HBV	C	-1,89	0,39	-1,04	0,74
				E	-1,64	0,34	-0,82	0,75
				HBcAg	-1,55	0,57	-1,27	0,74
				preC	-1,73	0,61	-0,82	0,56
	Retroviridae	Lentivirus	HIV-1	VPU	-Inf	1,05	-0,71	0,69
ssDNA	Parvoviridae	Dependoparvovirus	Adeno-associated virus	AAP	-0,92	0,83	-0,16	1,38
ssRNA-	Filoviridae	Ebolavirus	Bundibugyo ebolavirus	VP30	-1,91	0,36	-0,31	0,78
ssRNA+	Coronaviridae	Alphacoronavirus	NL63	E	-1,87	0,17	0,11	0,61
		Betacoronavirus	MERS	NS3A	-1,57	1,23	-0,24	0,90
				ORF3	-1,56	1,25	-0,22	0,89
			HKU1	M	-2,12	1,18	0,22	0,47
				N	-2,17	0,63	0,00	0,47
