# Supplementary material for: Footprint of the host restriction factors APOBEC3 on the genome of human viruses
Source: PLoS Pathog. 2020 Aug 14;16(8):e1008718. doi: 10.1371/journal.ppat.1008718 (PMC7449416; doi:10.1371/journal.ppat.1008718)
Supplement: S11 Fig — A. APOBEC1 favors cytidine deamination in a 5’ WCW context. The WCW trinucleotide motif is depicted in three possible codon contexts on both coding and template strand. Depending on the position of the mutated C, the C to T transition can be synonymous (S) or non-synonymous (NS). Proportion of S and NS mutations is reported when the two types of mutation can be produced. B. The NWCWNN and NWGWNN observed/expected ratios for 33,400 human viruses’ genomes (from 870 unique species) were calculated, grouped by species and colored according to the Baltimore classification. Each point represents a unique viral genome. C. The observed/expected ratios of WCW trinucleotide at various codon positions and on both strands (i.e. NWCWNN, WCW, NNWCWN, NWGWNN, WGW and NNWGWN) were calculated for the NWCWNN and/or NWGWNN depleted viral species and depicted by a heatmap. A colored scale with increasing shades of blue indicating depletion and increasing shades of red indicating enrichment. P-values were calculated by Student’s unpaired, two-tailed t-test (NS for not significant, * p< 0.05, ** p< 0.01, *** p< 0.001). D. List of the putative APOBEC1-footprinted viral genes (displaying NWCWNN or NWGWNN depletion) and belonging to an otherwise non-depleted viral genome. (PDF) [file ppat.1008718.s011.pdf]

Supplementary Figure 11

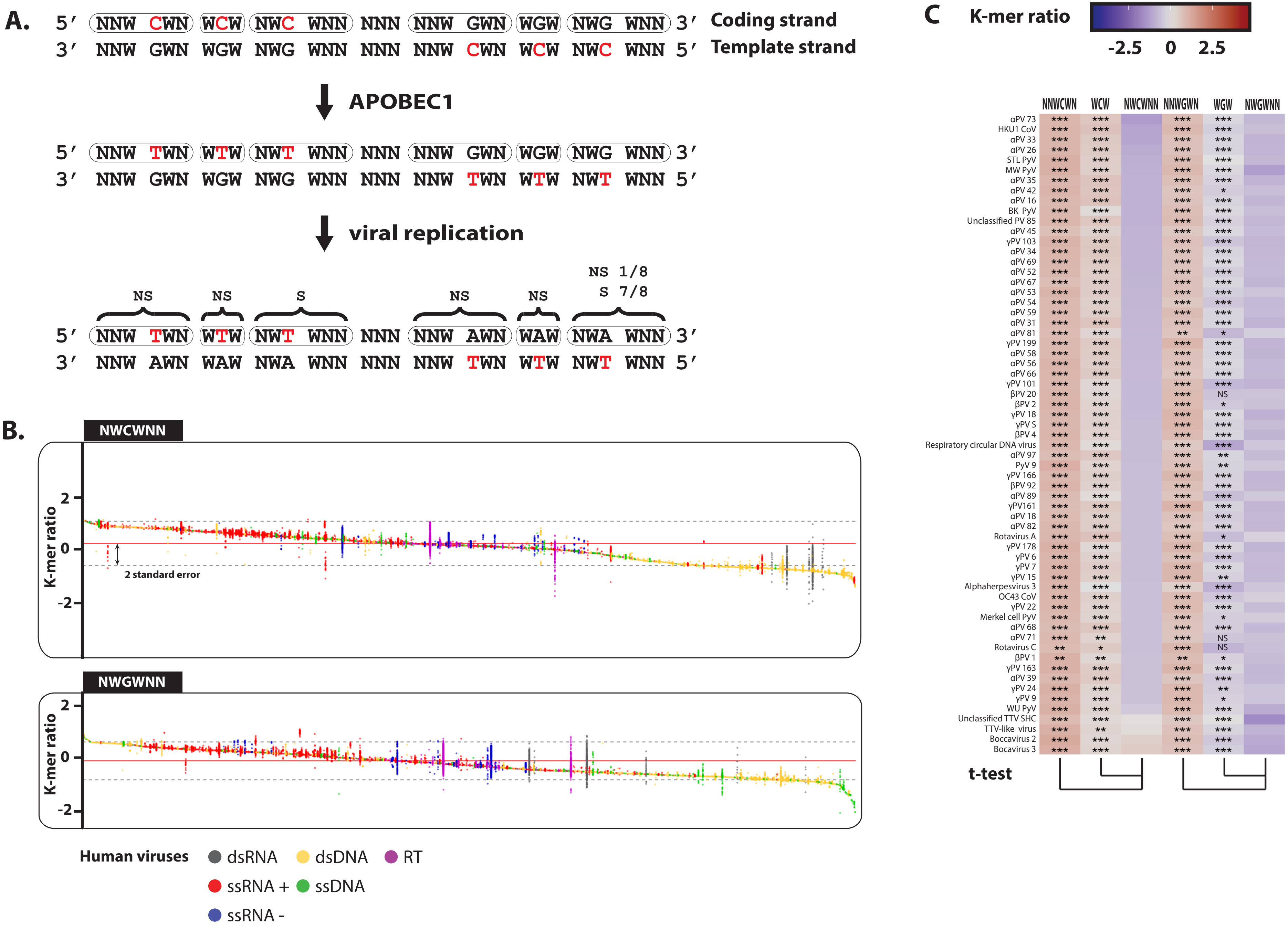

Supplementary Figure 11

D.

Group	Genus	Family	Specie	Gene	Genic NWCWNN	Genic WCW	Genic NNWCWN	Genomic NWCWNN
dsDNA	Adenoviridae	Mastadenovirus	Adenovirus B	E1A	-0,90	-0,45	-0,29	0,38
				E4	-0,83	0,03	0,19	0,37
				IX	-1,09	0,76	-0,44	0,37
			Adenovirus C	E1A	-0,84	-0,53	-0,45	0,46
				IX	-0,95	0,61	-0,43	0,46
			Adenovirus D	IX	-0,90	-0,60	-0,48	0,80
			Adenovirus E	E1A	-1,20	-0,79	-0,43	0,57
				IX	-0,75	0,27	0,26	0,57
			Adenovirus F	IX	-1,78	0,50	-0,21	0,19
	Herpesviridae	Simplexvirus	Alphaherpesvirus 1	US11	-0,69	-0,05	-0,11	0,76
				US8A	-0,66	-0,13	-0,11	0,76
			Alphaherpesvirus 2	UL14	-0,16	0,17	-0,15	0,96
				UL49A	-1,38	-1,39	0,80	0,96
				US10	-0,77	0,48	-0,24	0,96
				US11	-0,56	-0,90	-1,48	0,96
				US12	-0,26	-1,58	-0,11	0,96
				US2	-0,74	-2,16	-0,26	0,96
				US4	-0,18	-0,72	-0,02	0,96
				US5	-0,25	-0,81	0,51	0,96
				US8A	-1,39	0,19	-0,03	0,96
		Varicellovirus	Alphaherpesvirus 3	ORF25	-1,91	0,09	-0,32	-0,65
				ORF35	-2,43	-0,43	-0,05	-0,65
		Cytomegalovirus	Betaherpesvirus 5	UL80	-0,60	0,09	0,41	0,74
				RL5A	-1,25	0,82	0,11	0,74
				UL124	-0,50	1,04	0,44	0,74
				UL135	-0,37	-0,91	-0,25	0,74
				UL139	-0,55	1,06	-0,46	0,74
				UL146	-0,91	-1,04	0,13	0,74
				UL73	-0,75	1,16	-0,01	0,74
		Lymphocryptovirus	Gammaherpesvirus 4	BFRF3	-0,96	0,52	0,21	0,66
				BGLF3,5	-0,34	-0,37	0,66	0,73
				BHLF1	-3,24	-Inf	-2,23	0,66
				BLLF2	-1,61	-0,40	0,89	0,66
				BLRF2	-0,92	0,56	-0,65	0,66
				BSLF2	-0,82	0,21	0,78	0,80
				EBNA3A	-0,24	-0,25	0,57	0,80
				EBNA3B	-0,82	0,14	0,06	0,71
				LF3	-1,50	-2,24	-1,83	0,66
				RPMS1	-0,80	0,46	0,14	0,69
		Rhadinovirus	Gammaherpesvirus 8	K6	-0,90	-1,45	-0,45	0,42
				K7	-0,68	0,00	0,18	0,42
				ORF38	-1,16	-0,62	-1,12	0,41
				ORF73	-0,80	0,11	0,32	0,41
	Papillomaviridae	Alphapapillomavirus	PV type 31	E4	-Inf	0,67	0,69	-0,80
				E5	-2,56	0,02	0,72	-0,80
			PV type 33	E5	-Inf	-0,65	0,05	-1,15
			PV type 35	E5	-2,50	0,29	0,48	-0,93
			PV type 39	E5	-Inf	-0,69	-0,10	-0,61
			PV type 45	E7	-2,63	-0,36	0,51	-0,91
			PV type 51	E6	-2,00	-0,05	-0,23	-0,58
			PV type 56	L2	-2,85	0,97	-1,01	-0,76
			PV type 66	L2	-2,06	1,09	-0,86	-0,76
			PV type 73	E5	-Inf	0,09	0,41	-1,37
			PV type 82	E5	-2,51	0,12	-0,41	-0,67
				L2	-2,00	0,99	-0,75	-0,67
RT	Retroviridae	Lentivirus	HIV-1	TAT	-1,11	-0,06	0,38	0,05
			HIV-2	REV	-1,49	0,06	0,98	0,24
ssDNA	Parvoviridae	Dependoparvovirus	Adeno-associated virus	AAP	-0,42	1,36	-0,57	0,94
ssRNA+	Flaviviridae	Hepacivirus	HCV	F	-0,66	0,41	-0,11	0,92

Group	Genus	Family	Specie	Gene	Genic NWGWNN	Genic NNWGWN	Genic WGW	Genomic NWGWNN
dsDNA	Adenoviridae	Mastadenovirus	Adenovirus B	L5	-1,29	0,95	-0,40	0,19
			Adenovirus C	L5	-1,23	0,68	-0,94	0,30
			Adenovirus D	L5	-1,18	0,76	-1,43	0,55
			Adenovirus E	L5	-1,19	0,69	-0,98	0,49
	Herpesviridae	Simplexvirus	Alphaherpesvirus 1	UL1	-0,37	0,35	-0,39	0,85
				US11	-0,69	1,61	-0,30	0,86
				US12	-0,25	-0,27	0,19	0,86
			Alphaherpesvirus 2	UL1	-0,40	0,42	-0,84	0,96
				UL45	-0,19	-0,93	-0,22	0,96
				UL55	-0,56	-1,30	-0,55	0,97
				US11	-Inf	1,16	0,19	0,96
				US5	-0,17	-0,74	0,24	0,96
		Varicellovirus	Alphaherpesvirus 3	ORF13	-1,68	-0,05	-0,35	-0,52
				ORF9	-2,30	0,73	-0,60	-0,52
		Cytomegalovirus	Betaherpesvirus 5	RL1	-0,83	0,27	-3,43	0,55
				RL12	-0,83	0,60	-0,25	0,55
				RL13	-0,73	0,45	-0,35	0,55
				UL1	-1,06	-0,04	-0,11	0,55
				UL139	-0,88	-0,37	-0,24	0,55
				UL142	-0,82	-0,08	-1,08	0,55
				UL146	-0,89	0,04	-0,39	0,55
		Lymphocryptovirus	Gammaherpesvirus 4	BHLF1	-0,99	-1,99	-3,00	0,47
				BZLF2	-0,76	0,07	-0,13	0,47
				EBNA3A	-0,94	1,18	-1,56	0,64
				LF3	-0,89	-0,67	-1,89	0,47
				RPMS1	-1,95	-2,00	0,82	0,55
		Rhadinovirus	Gammaherpesvirus 8	ORF4	-0,78	0,77	-0,23	0,37
				ORF53	-1,03	0,20	-0,33	0,38
	Papillomaviridae	Alphapapillomavirus	PV type 16	L2	-4,88	1,03	-0,96	-0,84
			PV type 18	L2	-1,87	0,93	-1,30	-0,79
			PV type 30	L2	-1,74	1,05	-0,82	-0,62
			PV type 31	L2	-2,85	1,11	-0,59	-0,92
			PV type 33	L2	-2,24	1,19	-0,69	-0,89
			PV type 34	L2	-2,02	1,08	-0,44	-0,81
			PV type 35	L2	-2,36	0,96	-0,90	-0,91
			PV type 39	L2	-2,36	0,86	-0,97	-0,77
			PV type 53	L2	-1,95	1,15	-0,69	-0,55
			PV type 54	L2	-2,74	1,07	-1,24	-0,74
			PV type 6	L2	-2,26	0,91	-0,92	-0,76
			PV type 66	L2	-2,03	0,84	-0,97	-0,76
			PV type 68	L2	-2,11	1,00	-1,06	-0,79
			PV type 73	L2	-2,48	1,07	-0,21	-0,78
			PV type 82	L2	-1,96	0,91	-1,14	-0,81
sRNA-	Paramyxoviridae	Morbillivirus	Measles virus	V	-0,80	0,96	-1,55	0,51
ssRNA+	Coronaviridae	Betacoronavirus	OC43	NS2A	-1,72	1,17	-0,76	-0,34
				NS5A	-1,66	0,66	-0,09	-0,36
				NS4	-1,65	0,67	-0,16	-0,31
