# Supplementary material for: Footprint of the host restriction factors APOBEC3 on the genome of human viruses
Source: PLoS Pathog. 2020 Aug 14;16(8):e1008718. doi: 10.1371/journal.ppat.1008718 (PMC7449416; doi:10.1371/journal.ppat.1008718)
Supplement: Attachment — Submitted filename: 2020_05_29_Response to reviewers comments.pdf [file ppat.1008718.s016.pdf]

Resubmission for the manuscript “Footprint of the host restriction factors APOBEC3 on the genome of human viruses”

Dear Editors, dear Reviewers,

First of all, we shall thank you for considering our work and we are grateful for the time you spent on evaluating our manuscript.

This letter contains a detailed list of our responses to the review comments and a description of the changes we have made in the manuscript. Our detailed responses are colored in blue in the following text.

Reviewer's Responses to Questions

Part I - Summary
Please use this section to discuss strengths/weaknesses of study, novelty/significance, general execution and scholarship.

Reviewer #1: The manuscript “Footprint of the host restriction factors APOBEC3 on the genome of human viruses" by Poulain et al. expands upon previous work on classifying the depletion of APOBEC3 target motifs in over 24,000 human viruses and some non-human primate, avian, and fish viruses. Using methods adapted and expanded from Warren et al., the authors first validate that their analysis supports significant APOBEC3-motif depletion in polyomaviruses and papillomaviruses. They then identify that more viruses are depleted for this motif generally across their genomes. Additionally, some viruses that would normally not be considered depleted have significant depletion in certain regions of their genomes consistent with viral genome replication or transcription, such as adenoviruses and herpesviruses. This study is thorough and is an important comparison of APOBEC depletion using the same analysis approach across many virus families. There are some major concerns that need to be addressed first before I would recommend this interesting manuscript for acceptance.

Reviewer #2: To my knowledge this is the first valiant attempt to comprehensively analyze the APOBEC mutation signature across “all” viruses. This pan-virome analysis confirmed the documented presence of an APOBEC footprint on many viruses, most notably polyoma viruses and papillomaviruses. The approach also shed light on some less characterized APOBEC substrates including erythroviruses and coronaviruses (though curiously not those that recently zoonosed including human SARS-CoV-2). A gene-centric modification of the approach also indicated some interesting correlations with gamma-herpesvirus and adenovirus DNA replication strategies. Overall, the authors estimate an APOBEC impact on 16% of “all” viruses (though this number may be debatable because not all RNA viruses seem to be analyzed here).

Reviewer #3: Poulain and colleagues use a computational approach to identify a decrease in TC dinucleotides in a variety of viral genomes. The authors suggest that the decrease in TC, specifically NTC, represents an APOBEC3 footprint in these viral genomes. This has the

potential of being a really interesting story, but the authors need to address a couple of concerns before publication is warranted.

Part II – Major Issues: Key Experiments Required for Acceptance
Please use this section to detail the key new experiments or modifications of existing experiments that should be absolutely required to validate study conclusions.

Generally, there should be no more than 3 such required experiments or major modifications for a "Major Revision" recommendation. If more than 3 experiments are necessary to validate the study conclusions, then you are encouraged to recommend "Reject".

Reviewer #1: The taxonomic labeling of the supplemental tables is incorrect. The genus column contains family and subfamily classifications. The family column contains genus and the species column contains both species, type and strain. It is unclear how these discrepancies may have affected data analysis and plotting.

Additionally, I found an error in the annotation of one virus in the supplemental excel file. There may be more that could minorly affect the results:
ssRNA viruses Flaviviridae Alphapapillomavirus Tick-borne encephalitis virus

There were issues of taxonomic labeling in the Supporting Table file. Notably, there was a confusion between Family and Genus in the header of the excel file. There were also improper allocations of the Genus label for some particular viral species (e.g. Tick-borne encephalitis virus). The automatic procedure used to collect the Genus information for each given GenBank ID did not always collect the correct piece of information (depending on the formatting of the taxonomic information). We can reassure the reviewers that those taxonomic issues have not affected the data analysis and plotting. Indeed, we performed our analysis at the species level with an aggregation by Baltimore groups when necessary. There were no discrepancies between the GenBank ID and the corresponding species name and Baltimore group.
Importantly, for this revised version of the manuscript we chose to start over our analyses to use the new and curated “NCBI Virus” database (https://www.ncbi.nlm.nih.gov/labs/virus/vssi/#/).

We modified the Supporting Table files accordingly. The usage of this curated database did not modify any interpretation or conclusion made previously.

The authors should add more detail in the methods as to how the viral sequences were selected. Were identical sequences collapsed?

We downloaded complete viral genomes from the “NCBI Virus” database (https://www.ncbi.nlm.nih.gov/labs/virus/vssi/#/) as released in April 2020. We retrieved only full-length genomes by selecting “Complete” for the criterion “Nucleotide Completeness”. We retrieved Human viruses by selecting “Humans” for the criterion “Host”. We retrieved non-human primate viruses by selecting “Primate” for the criterion “Host” and by deducting the human viruses from this data set. We retrieved avian viruses by selecting “Aves (birds)” for the criterion “Host”. We retrieved fish viruses by selecting “Actinopterygii

(ray-finned fish)" for the criterion "Host". We also retrieved Camel MERS viruses by selecting "Camelus dromedaries (Arabian Camel)" for the criterion "Host" and "MERS-CoV" for criterion "Virus". We retrieved Bat MERS viruses by selecting "Chiroptera (bats)" for the criterion "Host" and "MERS-CoV" for the criterion "Virus". We retrieved Bat SARS viruses by selecting "Chiroptera (bats)" for the criterion "Host" and "Severe acute respiratory syndrome-related coronavirus" for criterion "Virus". The dataset of Human viruses was supplemented by manually curated human virus complete genome sequences from the "NCBI nucleotide" database. Using these criteria, 33,400 Human, 1,397 non-human primate, 9,160 avian, 570 fish, 259 Camel MERS, 5 Bat MERS and 33 Bat SARS full-length viral genomes were collected. GenBank accession ID's are treated as unique and listed in the S1, S2 and S3 Tables.

This paragraph has been added in the "Material and methods" section of the manuscript.

I'm not fully convinced that the Student's t-test of NTC/NNGANN versus TCN/NGA & NNTCNN/GAN is a necessary comparison. What is the biological take home message from these comparisons?

The goal of our analysis is to identify viral sequences displaying evidence for A3-driven natural selection. Because most A3 favor deamination of C in a 5'TC motif, we chose to measure the frequency of the TC motif in viral genomes.
A Cytosine to Uracil mutation will always be silent when the mutated C is at the third position of the codon. Therefore, we argue that A3-driven natural selection would deplete more intensively NTC codons than TCN or NNTCNN motifs (as in those cases the C to U mutation will impact the encoded amino acids). The Student's t-test of NTC versus TCN or NNTCNN intends to test the significance of these differences.
A significantly depleted NTC ratio vs non-depleted TCN and NNTCNN ratios would be interpreted as following: i. NTC depletion is tolerated up to a certain limit thanks to the degeneration of the genetic code; ii. the absence of TCN and NNTCNN depletion suggests that these viruses are still vulnerable to A3 restriction since deamination in those contexts would lead to non-synonymous mutations. These viruses are classified as A3-footprinted.
The distributions of the NNTCNN and TCN ratios also inform on the width of the distributions of TC-containing K-mers ratios within a given viral species. For example, the widths of the distributions of ratios are generally larger in dsRNA viruses than in dsDNA viruses.
Of note, most of viral species have a mean NTC ratio close to zero and not significantly different from the corresponding TCN or NNTCNN ratios. These viruses are classified as not A3-footprinted.
We never observed a viral species with strong and similar NTC, TCN and NNTCNN depletions.
The same logic applies for the NGA, GAN and NNGANN ratios which are instructive of deamination on the template strand.

In response to the reviewer's comments, we added:
lines 225-227 of the track changed manuscript:
"Therefore, A3-driven natural selection should deplete more intensively NTC codons than TCN or NNTCNN motifs (as in those cases the C to U mutation will impact the encoded amino acid)."

lines 265-270 of the track changed manuscript:

"The significant differences between the NTC ratio and the TCN (or NNTCNN) ratios reveal that the frequency of the TC motif is dependent on its position within a codon. It suggests that NTC depletion can be tolerated because of the degeneration of the genetic code. Importantly, the absence of NNTCNN and TCN depletion infers that this virus is still vulnerable to A3 restriction because deamination of those cytidines would lead to changes in the amino acid sequence."

The same logic applies for the putative A3G footprint:
lines 247-258 of the track changed manuscript:
"Secondly and because A3G favors deamination of cytosine to uracil in a 5'CC dinucleotide context, we have chosen to look for the under-representation of the 5'CC motif. Following the same rational, A3G-footprinted viruses should display to a stronger depletion of NCC codons compared to CCN or NNCCNN motifs (or a depletion of NNGGNN motifs versus the GNN and NGG motifs) (S1A Fig). The NCC ratio will be compared to those of the NNCCNN and CCN K-mers. Similarly, the NNGGNN ratio will be compared to those of the GGN and NGG K-mers."

lines 358-363 of the track changed manuscript:
"In order to specifically look for A3G-footprinted viruses, we calculated the NCC and NNGGNN ratios for 870 human viral species (S1B Fig). We observed that the NCC and NNGGNN ratios are mostly narrowly distributed around the zero value. Viruses depleted for NCC are generally single stranded DNA viruses (S1B Fig). However, in many of them we observed a concomitant depletion of the NNCCNN motif casting doubts on the causal link between the observed NCC depletion and A3G editing (S1C Fig)."

Reviewer #2: Major
1) At multiple points the authors claim to have analyzed "all" human viruses. This is obviously a moving target with many new viruses being reported each year (ex. thousands in one paper alone from a recent paper by the Buck lab – PMID 32014111 – which could alone change the results of the present study). Therefore, I suggest tempering this claim to "all currently annotated viruses" or all "NCBI references viruses" (and state the download window or dates in the methods).

Indeed, we fully agree and used the expression "all currently annotated human viruses". The corrections have been made throughout the manuscript.

2) Lines 190-92 indicate no depletion of A3G preferred NCC sites in viruses. A close look at Fig 3b shows some viruses with a clear underrepresentation of NCC sites. Are these retros? The authors should also comment on outliers that maybe impacted by A3G. In other words, the wholistic view is appreciated but a closer look at exceptions would also help balance the story.
3) Authors should extend analyses of gene-level footprints to include A3G-preferred motifs (in the very least as an additional negative control).

Because questions #2 and #3 are linked we formulated a global response.
We recognize that our first version of the manuscript was centered on the footprint of A3 proteins, excluding the A3G member. The reason for this apparent omission was that we did

not find convincing evidence of an A3G footprint neither at the genome nor at the genic level. We modified the manuscript at multiple instances to report these observations.

First, we formally stated our working definition of the A3G footprint.
Lines 247-258 of the track changed manuscript:
“Secondly and because A3G favors deamination of cytidine when preceded by another cytidine, we have chosen to look for the under-representation of the 5’CC motif. Following the same rational, A3G-footprinted viruses should display to a stronger depletion of NCC codons compared to CCN or NNCCNN motifs (or a depletion of NNGGNN motifs versus the GNN and NGG motifs) (S1A Fig). The NCC ratio will be compared to those of the NNCCNN and CCN K-mers. Similarly, the NNGGNN ratio will be compared to those of the GGN and NGG K-mers.”

Next, we broke down the K-mers ratios analysis of human viruses (Fig 4) into their respective Baltimore’s groups and generated the supplementary figure 3 (we particularly thank the reviewer for that suggestion, it clearly adds valuable information to the paper).
Lines 327-335 of the track changed manuscript:
“By breaking down the human viruses into their respective Baltimore’s group (S3 Fig), we observed that NTC depletion is not present in reverse transcribing nor in negative sense single strand RNA viruses. A mild general depletion is present in double strand RNA viruses. Importantly, a strong general depletion can be observed in double strand DNA viruses. Finally, in single strand DNA and positive sense single strand RNA viruses, certain specific viral sequences appear also significantly depleted. We also observed a mild general NCC depletion in single strand DNA and double strand RNA viruses, justifying further investigation for a possible A3G-induced footprint (S1 Fig). No NCC depletion is observed in double strand DNA, single strand RNA nor in reverse transcribing viruses.”

Because of a mild NCC depletion has been noticed in ssDNA and dsRNA viruses, we reported the search for genome-wide A3G footprint.
Lines 358-363 of the track changed manuscript:
“In order to specifically look for A3G-footprinted viruses, we calculated the NCC and NNGGNN ratios for 870 human viral species (S1B Fig). We observed that the NCC and NNGGNN ratios are mostly narrowly distributed around the zero value. Viruses depleted for NCC are generally single stranded DNA viruses (S1B Fig). However, in many of them we observed a concomitant depletion of the NNCCNN motif casting doubts on the causal link between the observed NCC depletion and A3G editing (S1C Fig).”

Then, we reported the search for gene-specific A3G footprint
Lines 540-544 of the track changed manuscript:
“An equivalent analysis was performed to report a local A3G footprint amongst an otherwise non-footprinted genome. NCC and NNGGNN-depleted genes are listed in Fig S1D. Similarly to what has been observed at the genome level, many of the NCC-depleted genes are also depleted for the NNCCNN motif making difficult to ascribe the observed NCC depletion to the sole A3G editing activity.”

4) To be as comprehensive as possible, the authors should also consider including the footprint of AID in this analysis, as the only family member preferring purine nucleobases on the 5' side of the target cytosine (RC).
We also extended our analysis for the search of an AID (target motif: WRC).
We did not observe any genome-wide AID-footprinted viruses. However, we identified local (gene specific) AID editing notably on herpesviruses.

The following paragraph has been added to the manuscript:
Lines 615-632 of the track changed manuscript:
"Search for AID or APOBEC1-footprinted viruses
The APOBEC family of genes also counts the AID (or AICDA), APOBEC1, APOBEC2 and APOBEC4 genes. AID is critical for somatic hypermutation and class switch recombination by editing the immunoglobulin loci in B cells [39]. APOBEC1 plays an important role in lipid metabolism by editing the apolipoprotein B pre-mRNA [40,41]. Importantly, APOBEC1 and AID appear also to participate to the restriction of viruses and retroelements [1,42]. Evidence for AID and APOBEC1 evolutionary footprints were investigated by looking for the depletion of their favored motifs, respectively WRC for AID [43] and WCW for APOBEC1 [44].
The distributions of the WRC and NNGYWN ratios do not point towards viruses significantly footprinted by AID at the whole genome level (S10B Fig). Nevertheless, putatively AID-footprinted genes were identified in several double strand DNA viruses, notably the B-cell-infecting virus EBV (S10C Fig).
The distributions of the NWCWNN and NWGWNN ratios show evidence of genome-wide footprinted viruses (S11B Fig). However, it is not possible to disentangle the APOBEC1 footprint from the APOBEC3 footprint as the 6-mers NWCWNN contains the 3-mers NTC. The putatively APOBEC1-footprinted viruses are those that also bear the putative APOBEC3 footprint (Fig 4B and S11C Fig).
Of note, APOBEC2 and APOBEC4-favored motifs have not been described so far."

5) HIV-1, HIV-2 and SIV should be have a dedicated results subsection and should be included in intraviral comparisons such as those in fig S6. The APOBECs were discovered as retrovirus restriction factors and they continue to have an impact on these viruses. The paper cites contrasting publications on the APOBEC footprint in retroviruses and I'm curious what the results are using the methodology described here in this ms.
We dedicated a result and a discussion subsection to HIV-1, HIV-2 and SIV. We reported a weak A3 evolutionary footprint on the negative strand of those viruses.

The following paragraphs have been added to the manuscript:
Lines 605-613 of the track changed manuscript – results section:
"Conflicting data were reported concerning the presence of an A3 evolutionary footprint on the HIV-1 genome [31,32]. Figure 11 reports ratios of the TC and GA-containing K-mers for the HIV-1, HIV-2 and SIV genomes. The HIV-1 genomes were spread out into their respecting groups (M, N, O) and subtypes (group M subtypes A, B, C, D and E). No NTC depletion was observed on the HIV-1, HIV-2 and SIV genomes (Fig 11). We concluded that A3s did not leave a footprint on the plus strand. Importantly, a mild but consistent NNGANN depletion was observed in HIV-1, HIV-2 and SIV genomes, depletion compatible with A3-editing during reverse transcription (Fig 11)."

Lines 1115-1122 of the track changed manuscript – discussion section:
"We paid particular attention to HIV in our analysis as the APOBEC3 antiviral activity has been historically discovered in that field of research [6]. We observed a weak A3 evolutionary footprint on the minus strand of the HIV genomes in support of the observations made by Jern et al. [31]. The weakness of the footprint can be explained at least in part by the efficiency of the A3-inhibiting protein Vif. Also, the error-prone RT could be responsible for the reversion of some A3-induced mutations providing that the virus can still complete its life cycle. Finally, A3s are well known to also restrict HIV through a deaminase independent activity, therefore without leaving any footprint."

6) A supplementary table of all downloaded file accession numbers should be included to enable full reproducibility.
We created a Supporting Tables S1, S2 and S3 containing the list of all downloaded viral genomes and viral coding genes (identified by their GenBank accession number). Along with each GenBank accession number, we report the calculated K-mer values. Tables S1 contains genomic K-mers ratios for the human viruses. Tables S2 contains genic K-mers ratios for the human viruses. Tables S3 contains genomic K-mers ratios for the non-human viruses.

Reviewer #3: 1. My main concern is related to how the 'synthetic genomes' are constructed by concatenating the different coding genes. It is not clear how overlapping genes are handled. It is clear that selection will work differently when 2 genes overlap. E.g. TCN in frame 1 = NTC in frame 2. However, a change in frame 2, will have a huge impact in frame 1 and will likely not be tolerated. The authors need to take this into account in their analysis. This is particularly important for the gene level analysis presented in figures 7, 8, 9 and 10. For example, is HBZ really affected by APOBEC, or is the apparent loss of TCs due to being the sole gene on the opposite strand, while being in an intron for 3 other genes?
We reckon that when genes overlap with reading frame offset, those genes are less likely to be footprinted by the A3 (i.e. display an NTC depletion). This is exactly what we observed in the erythroparvovirus B19 where the 7.5k gene somehow "protects" from A3 editing on the overlapping section of the NS1 gene (Fig. 5B). This example illustrates that the NS1 gene is NTC-depleted all along but not on the overlapping section with 7.5k gene. This phenomenon leads to a certain level of underestimation of the A3 editing. However, although we thought about it, we cannot think of a method that would discriminate for sure between a gene that is truly not footprinted versus a gene that is not footprinted because it overlaps with a second gene with a reading frame offset.
The case of the HBZ is interesting. We observe that the HBZ gene is depleted in NTC but not in NNGANN motifs. Thus, the loss of TC motifs in HBZ is strand specific. Because HBZ is an antisense transcript, NTC depletion of HBZ indicates that A3 editing occurs on the negative strand of the HTLV-1 genome. Similarly, the NNGANN depletion of the sense transcripts Gag, Pro, Pol and Tax also supports the A3 editing on the negative strand of the virus. Overall, these observations support that NTC depletion of HBZ gene is the true reflection of A3 editing activity.

2. I would like the authors to include a control for non-coding sequences. If the loss of NTC is truly correlated with the degenerate DNA code, one would expect that all TC, regardless of codon position, will be affected.

We calculated the NTC, TCN and NNTCNN ratios for non-coding sequences of HPV-16, 18 and 31. We added the plotted data on S5 Fig.

The following sentence has been added to the manuscript:
Lines 350-352 of the track changed manuscript:
"In the strongly NTC-depleted viruses HPV16, 18 and 31, a TC depletion is also observed in the non-coding region of the genome regardless of the analyzed motif (S5 Fig)."

3. APOBEC3 typically converts TC to TTT. The authors do not provide evidence that TC reduction is accompanied by an increase in TT. I would like to see this data. Specifically, in the case of the Coronaviridae where the evidence in favor of APOBEC mutations is, in my opinion, minimal (see next comment).
We calculated the NTT, NTA and NTG ratios for the putatively A3-footprinted viruses identified in Fig 4B and represented these data by a heat map (S4 Fig). We observed that the NTC depletion generally goes with a mild to a significative NTT enrichment. However, we do not think that we should necessary expect a strict conversion of NTC into NTT. Deamination of C to U would certainly lead to C to T mutations but not always. For example, in dsDNA viral genome, the BER pathway can convert the U to an abasic site and lead to another type of mutation. Also, the NTT that would result from NTC deamination can be converted to NTG or NTA by the viral error-prone polymerase along the numerous replication cycles.

Lines 349-350 of the track changed manuscript, we added:
"Of note, NTC depletion generally goes with a mild to a significative NTT enrichment (S4 Fig)."

4. The data in figure 4 is used to argue that in addition to dsDNA viruses, members of the Coronaviridae have reduced NTC content. The authors use two standard deviations around the mean of a bimodal distribution. The logic behind this choice is not completely obvious to me. It is clear that the average of this bimodal distribution is heavily skewed by a large group of viruses that are not TC depleted. However, there is minimal evidence that RNA is a substrate for APOBEC3. Indeed, there is more convincing evidence that APOBEC does not edit RNA. Therefore, the distribution is artificially skewed towards 0, which affects the validity of the 2xSD approach. I would like the authors to use a different approach to show that the TC depletion of HKU is real. Moreover, a recent paper showing that APOBEC3 restrict coronavirus replication, also showed no evidence of hypermutation upon overexpression of APOBEC, but points towards a non-canonical APOBEC function (1).

(1) 1) Milewska, A., Kindler, E., Vkovski, P. et al. APOBEC3-mediated restriction of RNA virus replication. Sci Rep 8, 5960 (2018). https://doi.org/10.1038/s41598-018-24448-2

It is true that the RNA editing activity of A3s (or of some A3) is still a matter of debate. However, there is a growing amount of evidence arguing in favor of such editing activity *in vitro* but also *in vivo*. The latest example is the presence of an A3 mutational signature in the positive single strand RNA Rubella virus (https://doi.org/10.1371/ journal.ppat.1008080).
Indeed, Milewska and colleagues' paper did not observe hypermutation, but crucially C to T and G to A point mutations were observed in HCoV-NL63 viruses passaged in A3-expressing cells but not in wild-type cells. The paper points toward A3 restriction of coronavirus replication by both deaminase-dependent and independent mechanisms.

Finally, a mutational spectrum compatible with APOBEC and ADAR editing was observed on the SARS-CoV-2 transcriptome, reinforcing the case for the RNA editing activity of A3 on coronaviruses (doi:10.1126/sciadv.abb5813).

To discuss the remarks raised by reviewer #3, we added the following paragraph:
Lines 806-820 of the track changed manuscript:
"However, recent reports demonstrated that A3A and A3G can deaminate ribocytidine within a single stranded RNA molecule [54–56]. In vivo, A3 mutational signature has been reported in the positive single strand RNA Rubella virus [57]. Importantly, Milewska and colleagues demonstrated that cytoplasmic A3s can restrict the NL63 coronavirus in vitro [19]. The A3-mediated restriction of the HCoV-NL63 appears to be both deaminase-dependent and independent. A3 restriction did not cause hypermutation on the viral genome, but C to T and G to A point mutations were observed in HCoV-NL63 viruses passaged in A3-expressing cells but not in wild-type cells. It is a matter of debate whether the hypermutated genomes could not be retrieved because of the high fitness cost of such mutations or because the A3 are less processive on coronaviral RNA. Additionally, A3 proteins have been shown to interact with the nucleoproteins of the HCoV-229E, HCoV-NL63 and SARS-CoV-1 [19,58]. Finally, a recent report demonstrates the presence of APOBEC and ADAR editing on the SARS-CoV-2 transcriptome [59]. Thus, knowing that A3s can bind the nucleoprotein and that A3 footprint is present on the positive strand of the viral genome, we suggest that A3 editing occurs on the packaged genome."

5. The authors suggest a difference between human CoV and zoonotic CoVs. The authors need to take the evolutionary history of these viruses into account.
We thank the reviewer for this advice and add the following paragraph to the manuscript:
Lines 820-832 of the track changed manuscript:
"Two beta-coronaviridae are endemic to humans (HCoV-OC43 and HCoV-HKU1), they are widespread, have been circulating in human for at least several decades and may cause 10 to 15% of common colds (review in [60]). Both have an A3 footprint on the positive strand. In comparison, no evidence of footprint was observed on the zoonotic beta-coronaviridae SARS-CoV-1, MERS-CoV or SARS-CoV-2. The absence of an evolutionary footprint on SARS-CoV-1 and MERS-CoV could find its explanation in the relative low number of infected individuals and the short duration of viral circulation. According to the World Health Organization, SARS-CoV-1 infected about 8.000 people over a period of few months and have been declared eradicated in May 2004. The MERS-CoV infected so far less than 3.000 people by causing episodic outbreaks in the Middle East. The figures for the SARS-CoV-2 are radically different with more than 5 million confirmed cases as of May 2020. In that respect, it will be interesting to track the evolution of the pandemic SARS-CoV-2 regarding a possible introduction of an A3 footprint through its interhuman transmissions."

Furthermore, bats have a significantly expanded APOBEC repertoire compared to humans, so the authors need to explain why bat APOBEC would not edit the CoV genomes, but human APOBECs do.

Although we can only speculate, we discussed this issue by adding the following paragraph to the manuscript:
Lines 832-841 of the track changed manuscript:

“It is worth reiterating that no footprint was detected on the SARS bat isolates, although the bat A3 locus is the largest and most diverse known repertoire of A3 genes in mammals [61]. Perhaps SARS-like viruses possess a yet unknown and unique A3 inhibiting mechanism. Interestingly, the SARS-CoV-2 genome contains a novel ORF, called ORF10. ORF10 encodes a protein that has been demonstrated to interact with the CUL2 complex [62]. This interaction is reminiscent of the interaction between BIV Vif and CUL2 [63]. One could speculate that ORF10 could play a Vif-like role in bat and also in Human. Also, a shorter version of SARS-CoV-2 ORF10 is present in all SARS-like viruses [64]. This could explain why the SARS-like viruses are not A3-footprinted.”

Part III – Minor Issues: Editorial and Data Presentation Modifications
Please use this section for editorial suggestions as well as relatively minor modifications of existing data that would enhance clarity.

Reviewer #1: Line 100: this should be tempered to be representatives from all known human virus families. The authors do not have all papillomavirus types (400+) and therefore cannot claim all viruses.
Indeed, we fully agree and used the expression “all currently annotated human viruses”. The corrections have been made throughout the manuscript.

Considering the previous point, the claim that 16% of all viruses being A3-motif depleted may be incorrect. How was this calculated initially?
Among the 870 human viral species tested for A3 footprint, 143 (16.7%) shown significant NTC depletion and 136 (15.6%) significant NNGANN depletion. In total, 175 species (22.4%) present an A3 footprint on either one or both strands. These figures are based on the updated sequence dataset totalizing now 33,400 viral sequences.

Figure 9. Is RPMS1 completely depleted or is this an error? The other ORFs that are labeled black in panel A should be a color that is not on the k-mer ratio color scale.
RPMS1 is completely depleted for NTC motif and is therefore colored in black.
We modified the color of the genes’ arrows in Fig 9 panel A to match the color scale.

Lines 370-378: Non-human coronaviruses do not show a TC depletion signature, but do they show evidence for deamination by bat APOBECs? Bat retroviruses show more mutation for GG to AG rather than GA to AA according to Hayward et al. Mol. Biol. Evo. 2018.
Unfortunately, the RPo assay used in that paper doesn’t comprehensively test all C containing trinucleotides, so there is not a clear motif preference for each bat APOBEC3, but could be potentially inferred by expanding the motif analysis beyond TC.
We extended our motif analysis to study the frequency of the 5’CC motif.
Although we did not add that figure to the manuscript, we show below the search for a possible 5’CC motif depletion on human and zoonotic coronaviruses.
Nevertheless, we did not observe any NCC nor NNGGNN significant depletion that would be indicative of a 5’CC to 5’CT editing activity.

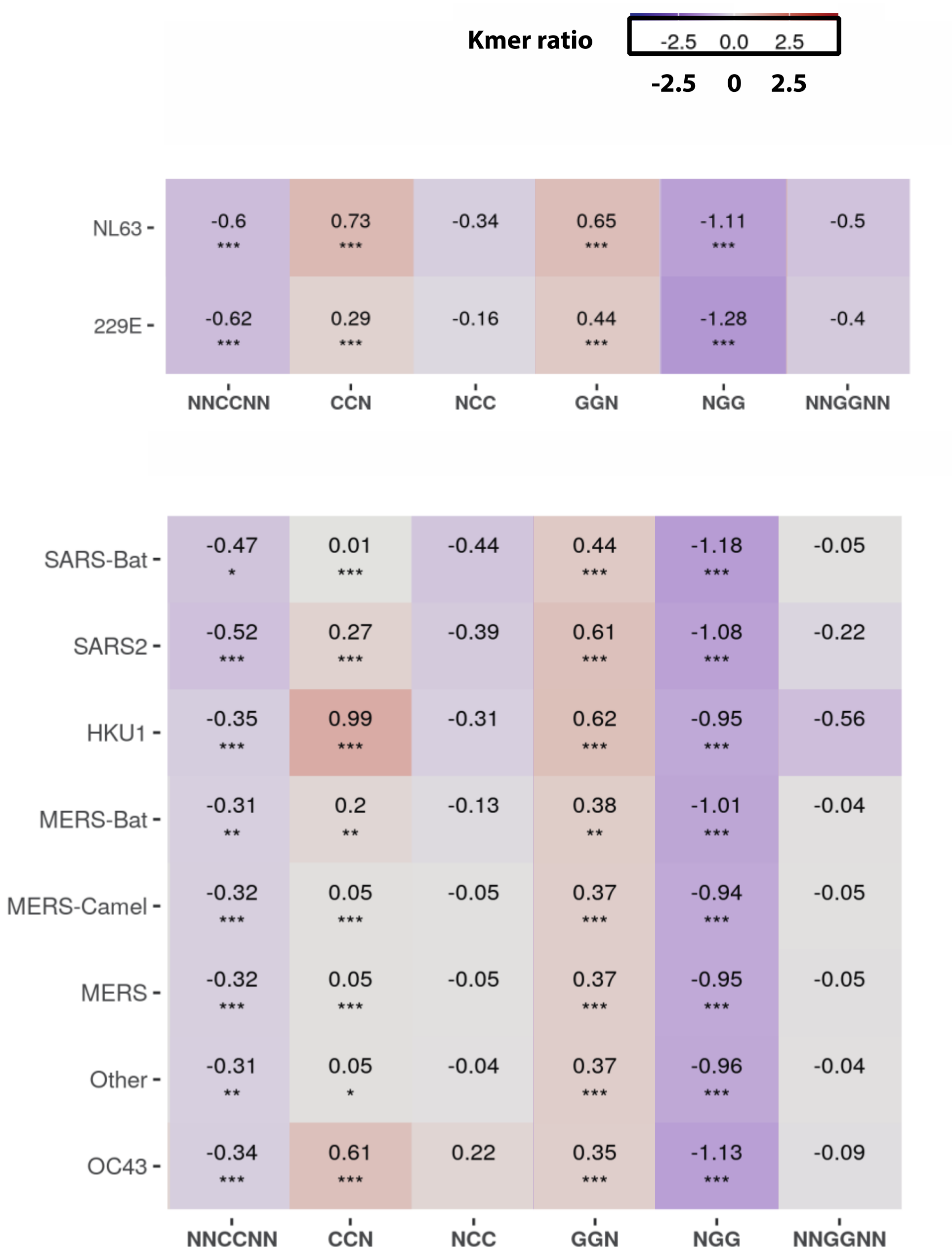

The search for evidence of 5'CC to 5'CT editing activity (putative A3G footprint) has been added to the manuscript:

Lines 358-363 of the track changed manuscript:

"In order to specifically look for A3G-footprinted viruses, we calculated the NCC and NNGGNN ratios for 870 human viral species (S1B Fig). We observed that the NCC and NNGGNN ratios are mostly narrowly distributed around the zero value. Viruses depleted for NCC are generally single stranded DNA viruses (S1B Fig). However, in many of them we observed a concomitant depletion of the NNCCNN motif casting doubts on the causal link between the observed NCC depletion and A3G editing (S1C Fig)."

Lines 540-544 of the track changed manuscript:

"A similar analysis was performed to report a local A3G footprint amongst an otherwise non-footprinted genome. NCC and NNGGNN-depleted genes are listed in Fig S1D. Similarly to what

has been observed at the genome level, many of the NCC-depleted genes are also depleted for the NNCCNN motif making difficult to ascribe the observed NCC depletion to the sole A3G editing activity."

In a similar vein to the previous comment, is it possible that APOBEC1 (target motif: YCY) is the primary DNA deaminase against viruses in the absence of APOBEC3 enzymes? What about AID (WRCN)?

We also extended our analysis for the search of an AID (target motif: WRC) or APOBEC1 (target motif: WCW).

We did not observe genome-wide AID-footprinted viruses. However, we identified local (gene specific) AID editing notably on herpesviruses. The search for A1-footprinted virus was more complicated as the NWCWNN 6-mers contains the NTC 3-mers. Thus, using our approach, the A1 footprint cannot be disentangled from the A3 footprint.

The following paragraph has been added to the manuscript:

Lines 615-632 of the track changed manuscript

"Search for AID or APOBEC1-footprinted viruses

The APOBEC family of genes also counts the AID (or AICDA), APOBEC1, APOBEC2 and APOBEC4 genes. AID is critical for somatic hypermutation and class switch recombination by editing the immunoglobulin loci in B cells [39]. APOBEC1 plays an important role in lipid metabolism by editing the apolipoprotein B pre-mRNA [40,41]. Importantly, APOBEC1 and AID appear also to participate to the restriction of viruses and retroelements [1,42]. Evidence for AID and APOBEC1 evolutionary footprints were investigated by looking for the depletion of their favored motifs, respectively WRC for AID [43] and WCW for APOBEC1 [44].

The distributions of the WRC and NNGYWN ratios do not point towards viruses significantly footprinted by AID at the whole genome level (S10B Fig). Nevertheless, putatively AID-footprinted genes were identified in several double strand DNA viruses, notably the B-cell-infecting virus EBV (S10C Fig).

The distributions of the NWCWNN and NWGWNN ratios show evidence of genome-wide footprinted viruses (S11B Fig). However, it is not possible to disentangle the APOBEC1 footprint from the APOBEC3 footprint as the 6-mers NWCWNN contains the 3-mers NTC. The putatively APOBEC1-footprinted viruses are those that also bear the putative APOBEC3 footprint (Fig 4B and S11C Fig).

Of note, APOBEC2 and APOBEC4-favored motifs have not been described so far."

Potentially out of the scope of this paper, but I am curious whether any mouse viruses show mouse APOBEC3 motif depletion (TYCN)?

We analyzed 256 sequences of mouse viruses and calculated TYC ratio. The results are illustrated below (we did not include that figure in our manuscript as it was slightly out of scope). Several parvoviruses and coronaviruses show a significant depletion for the TYC K-mer.

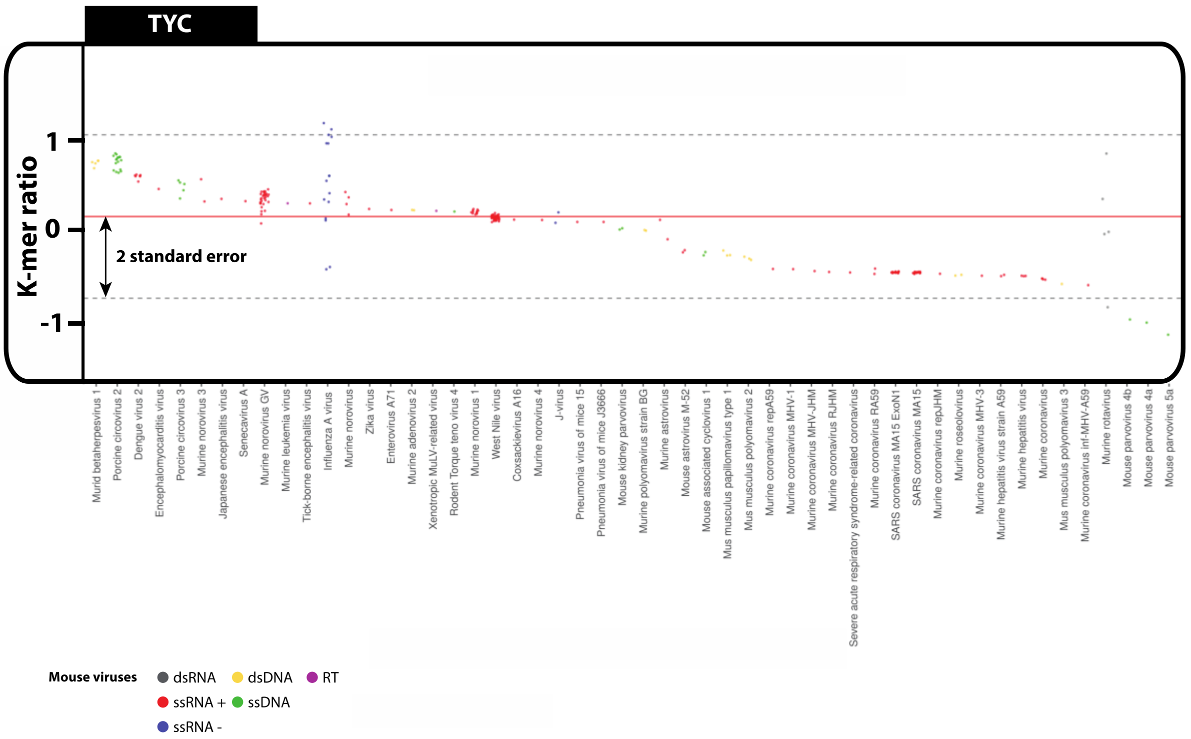

The sentence on line 249 is unclear: "Nevertheless, the observation of a general depletion of the NGA codons precludes any conclusion regarding the presence of an A3 footprint on the negative strand (Fig 6)"

We removed that sentence and replaced it by the following:

Lines 456-461 of the track changed manuscript:

"Next, we investigated the presence of an A3 footprint on the template strand (corresponding to the negative strand in coronaviruses) by comparing the 5'GA ratios in different codon contexts. However, we did not observe a progressive depletion of the GA motif (i.e. NNGANN ratio < NGA ratio ≤ GAN ratio) which would be expected in the presence of a GA to AA mutational pressure. For that reason, we cannot conclude in favor of the presence of an A3 footprint on the negative strand (Fig 6)."

I completely agree that NTC/NNGANN is a benchmark for APOBEC3 activity on viruses over evolutionary time. Can the authors speculate on whether viruses with depletions in TC/GA motifs that would otherwise result in coding changes are more protected against APOBEC3 activity and therefore in less need of anti-APOBEC proteins such as Vif or BORF2.

In the most intensively A3-footprinted viruses (e.g. *papillomaviridae* or *polyomaviridae*) and despite the very low NTC and NNGANN ratios, the TCN/NNTCNN and GAN/NAG motifs were not found to be depleted. Those viruses are therefore still susceptible to A3 restriction. Indeed, deamination of the remaining TCs would be most of the time non-synonymous. Compared to large DNA viruses like the *herpesviridae* equipped with anti-APOBEC protein, the small size of the *papilloma*- and *polyomaviridae* genomes could allow these viruses to cope with a high mutation rate. We speculate that the strong depletion of the NTC and NNGANN motifs observed in the *papilloma*- and *polyomaviridae* reduces exposure of the viral genome to the introduction of uracil and therefore to the base excision repair-mediated DNA degradation.

We introduced these ideas at different places in the manuscript:
Lines 702-706 of the track changed manuscript:
“we show that the papillomaviridae and the polyomaviridae families are those whose footprint is most intensive (Fig 4A). Those viruses have evolved to thrive under an ongoing and strong A3 selection pressure. The strong NTC depletion reduces exposure of the viral genome to the introduction of uracil and therefore to the base excision repair-mediated DNA degradation.”

Lines 713-719 of the track changed manuscript:
“On the one hand, A3A has been shown to restrict HPV in vitro [45]. Those viruses are still susceptible to A3 restriction. Indeed, deamination of the remaining TC motifs are most of the time non-synonymous. On the other hand, the deaminase activity could positively impact viral fitness by participating to the genetic diversification of the virus or even by protecting the host cell against the reactivation of retroelements [50,51]. We speculate that the error rate of the host DNA polymerase is too low for viruses with such small DNA genome, hence requiring the A3 editing activity to drive their evolution.”

Lines 1041-1045 of the track changed manuscript:
“The strategy deployed by EBV and KSHV to cope with A3 restriction is somehow opposite to the one used by the papilloma and polyomaviruses. Indeed, EBV and KSHV actively protect their genome from the A3s as opposed to the papilloma and polyomaviruses which simply cope with a high mutational rate. We speculate here that the large size of the herpesvirus genome would not tolerate an unrestrained A3 activity.”

Parvovirus B19 also perturbs the cell cycle and E2F family of genes through NS1 (PMID: 20890043, PMID: 282640280). For BKPyV (and likely relatives) this function seems to be essential for APOBEC3B upregulation. Does this differ from other parvoviruses as a potential explanation for the observed APOBEC3 motif depletion?
The literature on human parvoviruses other than the erythroparvovirus B19 is still scarce and does not allow us to speculate any further on the reason why the A3 footprint is so intense in the erythroparvovirus B19. Indeed, it would be very interesting to test whether B19 NS1 triggers or favors A3B expression in vitro and to compare with the NS1 from the bocaviruses.

Do the codons in the Parvovirus B19 genome that overlap the 7.5k protein encode conserved amino acids for function or are these diverse?
The NS1 codons that overlap the 7.5k protein are conserved and notably contain the transactivation domain TAD2 responsible for NS1-induced G2-phase arrest (Parvovirus B19 NS1 protein induces cell cycle arrest at G2-phase by activating the ATR- CDC25C-CDK1 pathway; Xu et al.; DOI:10.1371/journal.ppat.1006266). The 7.5k protein has not been thoroughly studied so far and functional conserved domains have not been described yet.

Line 358: The sentence “This observation came as a half surprise." Is unnecessary.
This sentence has been removed. That was indeed unnecessary.

References 34 and 38 out of order in the text.
The references have been reworked.

428: edition should be editing
The mistake has been corrected.

Reviewer #2: Minor
- The animal pictures are not necessary in Fig 6.
The animal pictures have been removed.
- Line 363 – “Parallelly” is not a word; try “In parallel” instead.
The mistake has been corrected.
- Line 428 – “edition” is not a word; use “editing” instead.
The mistake has been corrected.
- Nomenclature – use accepted names recent zoonotic virus - SARS-CoV-2 or COVID-19; not the much less common “Wuhan seafood market pneumonia virus”).
The expression Wuhan seafood market pneumonia virus has been removed and replaced by SARS-CoV-2.
- The references are not yet in the correct format for PLoS Pathogens (and some like #5 and #11 and others are incomplete)
The references were reordered and formatted using the PLoS Pathogens template.

Reviewer #3:
1. The authors should consider moving Figure 1 and the associated paragraph moved to materials and methods.
The PLoS pathogens template requires the Material and Methods section being at the end of the manuscript. Thus, we prefer to keep Figure 1 and the associated paragraph at the beginning for simplicity of reading.

2. Overall the figures are overly complicated. I do not feel like they really convey the results in an optimal manner. I would urge the authors to use less figures and improve their interpretability.
Data representation is indeed a major difficulty. We paid attention to avoid unnecessary colors or details but it has been overall very challenging to fundamentally change the figures.

3. Figure 2, The authors conclude that both strands are edited. It is however clear that NTC is significantly less common than NNGANN. This should be addressed.
Indeed, we first made it clear in the material and methods section that the 3 letters motifs include 4 codons whereas the 6 letters motifs include 256 pair of codons.
We made it clear by adding lines 1280-1282 in the material and method section:
“For example, the NTC K-mer actually includes the ATC, CTC, GTC and TTC codons. Following the same logic, the NNTCNN K-mer comprises the 256 pair of codons having a T at the end of the first codon and a C to start the second codon.”

We express the abundance of a given K-mer as a ratio between the number of observed occurrences over the number of expected occurrences. This corrects for the difference of absolute abundance between the 3-mers and 6-mers.

4. The authors compare human viruses to viruses in other groups of organisms. The authors should do this comparison for individual Baltimore classes. However, given the data in figure

4, it may not be relevant to compare anything but dsDNA viruses. Figure 3 should include some sort of statistical support.
We broke down the K-mers ratios analysis of human viruses (Fig 4) into their respective Baltimore's groups and generated the supplementary figure 3.
We detailed these results on lines 327 to 335 of the track changed manuscript:
"By breaking down the human viruses into their respective Baltimore's group (S3 Fig), we observed that NTC depletion is never observed in reverse transcribing nor in negative sense single strand RNA viruses. A mild general depletion is present in double strand RNA viruses. Importantly, a strong general depletion can be observed in double strand DNA viruses. Finally, in single strand DNA and positive single strand RNA viruses, certain specific viral sequences appear also significantly depleted. We also observed a mild general NCC depletion in single strand DNA and double strand RNA viruses, justifying further investigation for a possible A3G-specific footprint (S1 Fig). No NCC depletion is observed in double strand DNA, single strand RNA nor in reverse transcribing viruses."

As no NTC depletion has been observed in fish or avian viruses, we did not add dedicated figures for each Baltimore groups.

To avoid overloading of the Fig 3, we kept statistical comparisons for the heat map on Fig 4.
